# Investigation of regioselectivity and thermostability of free and immobilized *Pleurotus citrinopileatus* lipase

**DOI:** 10.1007/s00253-026-13801-5

**Published:** 2026-04-07

**Authors:** Lea Henrich, Jan Passinger, Moritz Nawrath, Foteini Batsolaki, Apostolos Spyros, Ioannis V. Pavlidis, Binglin Li, Martin Gand

**Affiliations:** 1 Institute of Food Chemistry and Food Biotechnology, Justus Liebig University Giessen, Giessen, Germany; 2 Department of Chemistry, University of Crete, Heraklion, Greece; 3 College of Food Science and Engineering, Northwest University, Xi’an, Shaanxi China; 4 Institute of Food Science and Technology Nutrition and Health (Cangzhou), Chinese Academy of Agricultural Sciences, Cangzhou, China

**Keywords:** Fungal lipase, Immobilization, Thermostability, Regioselectivity, Protein engineering

## Abstract

**Abstract:**

A fungal lipase from the Basidiomycete *Pleurotus citrinopileatus* (PCI_Lip) has been investigated for its regioselectivity. Therefore, the conversion products of a triglyceride were analyzed using ^1^H-NMR to determine the position of hydrolysis within the substrate. PCI_Lip is a 1,3-selective lipase, as it preferentially hydrolyzes the fatty acids at the outer positions of the triglyceride. Furthermore, this study aimed to gain deeper insights into the stability of the lipase. The melting point of the enzyme was determined to be at 63 °C in ultrapure water, which could be increased to 71 °C by dissolving PCI_Lip in a 400 mM citrate buffer pH 6.0 with the addition of glucose and trehalose. To optimize the stability of the enzyme, PCI_Lip was immobilized via its affinity tag. Twenty-one supports were tested, with IB-HIS-19 being chosen as the most appropriate for PCI_Lip immobilization due to its high binding capacity and low leakage. This immobilized lipase displayed only minor loss of activity over 13 cycles of reuse. Immobilization of the wild-type preserved activity and selectivity, whereas it induced notable changes in the case of the double mutants. The increased stability of the immobilized PCI_Lip was further proven in a thermal inactivation experiment. Double mutants of PCI_Lip displayed even higher inactivation temperatures, while the immobilization raised this temperature by 5 °C. Overall, in this study, the 1,3-regioselective PCI_Lip identified from our group has been further characterized, focusing on the improvement of its thermal and kinetic stability. A major boost in stability can be achieved via immobilization of the enzyme to an appropriate support.

**Key points:**

- *IB-HIS-19 had the highest binding capacity and kept up enzyme activity over 13 cycles of the screened immobilization materials*
- *Immobilization increased the stability of PCI_Lip by 5 °C, protein engineering increased the stability of two double mutants up to 4 °C, and thermostability could be increased by 9 °C by using high molarity buffers*
- *PCI_Lip was found to be a 1,3-regioselective lipase by analyzing its products by *^*1*^*H-NMR*

**Supplementary Information:**

The online version contains supplementary material available at 10.1007/s00253-026-13801-5.

## Introduction

Various industries such as food, pharmaceutical, and chemical industry are shifting towards more sustainable production systems and in this effort, enzymes have been applied as biocatalysts in different production processes. Biotechnological processes operate under mild reaction conditions in terms of temperature and pressure (Iyer and Ananthanarayan 2008; Silva et al. 2018; Stepankova et al. 2013). For these applications, recombinant protein expression is used to efficiently produce large quantities of enzymes with up to 50% of the host’s protein metabolism dedicated to heterologous expression (Zhao et al. 2024). However, overexpression bears the risk of low solubility of the protein, loss of activity due to conformational changes, enzyme aggregation, or even precipitation (Boivin et al. 2013; Silva et al. 2018). Selecting appropriate buffer components can affect enzyme solubility and stability and help prevent the formation of inclusion bodies by supporting proper folding of the enzyme. While kosmotropic ions support enzyme stabilization, chaotropic ions destabilize the dissolved enzymes (Ugwu and Apte 2004). The addition of neutral salts can help with stabilization as they interact with the charges on the protein surface and thus support hydrophobic interactions within the macromolecule. Stabilizing as well as destabilizing effects of neutral salts are closely related to the Hofmeister series (Silva et al. 2018). Moreover, the thermostability can be further enhanced by the addition of sugars and polyols. These substances form a layer around the macromolecule that can only be passed by water molecules, which results in an improved hydration (Silva et al. 2018).

However, optimization of the storage buffer in most cases is insufficient to adapt enzymes to the requirements of industrial processes. Most enzymes do not meet the requirements for direct application in industrial operations, as these reactions do not occur under physiological conditions. Enzymes applied in industry often face challenges such as high temperatures, extreme pH conditions, high salinity, and also organic solvents which lead to denaturation (Iyer and Ananthanarayan 2008). Immobilization offers stabilization of the enzyme in such environments. Immobilized enzymes can also be separated from the reaction mixture, enabling the accurate control of the process. Another advantage is their reusability, as immobilized enzymes can be applied in several reaction cycles. The chance to remove the enzyme from the products makes this technique particularly interesting for applications in the food industry, as no listing as a food ingredient is required (Yushkova et al. 2019; Sheldon 2007). Enhanced stability of immobilized enzymes is attributed to the increased rigidity of the biocatalyst (Stepankova et al. 2013). Adsorption of the enzyme to the support is the easiest way of immobilization, though binding is not strong and leakage is high. Covalent binding using bifunctional spacers attaches the enzyme sturdier to the support. It offers good immobilization yields with low leakage, but bears the risk of loss of activity when the biocatalyst is immobilized in a way that the substrate cannot enter the active site (Homaei et al. 2013; Maghraby et al. 2023; Silva et al. 2018; Stepankova et al. 2013; Sheldon 2007). Encapsulating the enzyme into a polymer matrix and cross-linking of enzymes are further methods to immobilize biocatalysts (Homaei et al. 2013; Sheldon 2007). A specific way to immobilize enzymes onto a support is affinity interaction. This strategy makes use of tags originally fused to the enzyme as purification tags. The commonly used histidine tag is used to bind to a chelate-bound transition metal. For this immobilization method, the support is functionalized with chelating moieties (Homaei et al. 2013).

Hydrolases, particularly lipases (EC 3.1.1.3), are of major interest for industry. Their chemo-, regio-, and enantioselectivity and also their wide substrate spectrum make them a sustainable substitute in chemical production processes. Depending on the process conditions, these enzymes can catalyze hydrolysis, esterifications, transesterifications, acidolysis, or aminations. Another benefit is that hydrolases do not require a cofactor (Abellanas-Perez et al. 2024; Stepankova et al. 2013). In this work, we used a lipase from the Basidiomycete *Pleurotus citrinopileatus* (PCI_Lip). In a previous work of our group, the lipase has been used as a substitute for pregastric esterases, enzymes used in cheese production to generate flavor by the release of volatile free fatty acids (Sowa et al. 2022). As the amount of long-chain fatty acids released by the enzyme caused a slightly soapy off-flavor in the prepared cheese samples, a mutant library has been generated. In a semi-rational design approach, optimization of the hydrolysis profile of the enzyme was conducted (Broel et al. 2022). In this study, we combine the Basidiomycota-derived lipase with affinity immobilization to enhance the stability of PCI_Lip for putative industrial applications. Another aspect was the investigation of the regioselectivity of PCI_Lip. Natural triglycerides often carry different fatty acid moieties at the three ester positions. These triglycerides are chiral molecules with a stereo-specific numbering of *sn* 1–3 for the different positions of the fatty acid esters. Lipases are categorized into nonspecific lipases, hydrolyzing any ester bond within a triglyceride, and regiospecific lipases only active on the two outer ester bonds (Park and Park 2022). All in all, this study gives insights into improved conditions and reactivity for a fungal lipase for an expanded field of applications in industrial processes.

## Material and methods

### Enzyme production and purification

Heterologous expression of PCI_Lip wildtype (WT) and the two double mutants S163M+L302G and I245F+L302G was performed according to Henrich et al. (2026). The gene of PCI_Lip originates from *P. citrinopileatus* (DSM number 5341, GenBank file OL364849). Briefly, *Escherichia coli* pellets from 200 mL culture were lysed by sonication, purified by immobilized metal ion affinity chromatography (IMAC), and desalted. Purified lipase was stored at 4 °C and expression was checked via sodium dodecyl sulfate polyacrylamide gel electrophoresis (SDS-PAGE) (Broel et al. 2022; Laemmli 1970).

### Hydrolytic activity

Lipolytic activity of free and immobilized PCI_Lip was tested in photometric assays using *para*-nitro phenyl (*p*NP) fatty acid esters according to Broel et al. (2022). The model substrates were *p*NP-acetate (*p*NPA) (Sigma-Aldrich, Taufkirchen, Germany), *p*NP-butyrate (*p*NPB) (Th. Geyer, Renning, Germany), *p*NP-valerate (*p*NPV) (Sigma-Aldrich), *p*NP-hexanoate (*p*NPH) (TCI Europe, Heuchelheim, Germany), *p*NP-octanoate (*p*NPO) (Alfa Aesar, Karlsruhe, Germany), and *p*NP-palmitate (*p*NPP) (Sigma-Aldrich). One unit of enzyme activity is defined as the amount of enzyme that produces 1 µmol of *p*NP in 1 min in the respective assays. Protein concentration was determined according to the method of Bradford (Bradford 1976; Broel et al. 2022). To examine the hydrolytic activity of immobilized enzyme, Triton X-100 was substituted with the same amount of dimethyl sulfoxide (DMSO) (Honeywell, Seelze, Germany) with *ε*_*p*NP_ = 0.0076 L ∙ µmol^−1 ^∙ cm^−1^. Statistical evaluation in this and the following experiments was performed by one-way analysis of variance (ANOVA) with a significance level of 0.05 in OriginPro 2023 (OriginLab, Northampton, USA).

### Enzyme immobilization

For enzyme immobilization, 17 IB-HIS carriers (ChiralVision, Den Hoorn, Netherlands) (Supplementary Table S1) and four carriers from Purolite (Ratingen, Germany) were tested. Adsorptive immobilization was performed by adding 1 mL of enzyme solution to 5 mg of carrier. Immobilization was performed at 800 rpm, 4 °C for 22 h. For covalent immobilization, 125 mg of carrier was mixed with 1 mL of 0.125% glutardialdehyde (Fisher Scientific, Schwerte, Germany) solution and incubated at 650 rpm, 25 °C for 2 h. The supernatant was discarded, and the carrier was washed with 1 mL 50 mM potassium phosphate (Carl Roth, Karlsruhe, Germany) buffer (PPB) at 650 rpm, 25 °C for 30 min. Supernatant was discarded again. Five milligrams of the activated carrier was then incubated with 1 mL of enzyme solution at 800 rpm, 4 °C for 22 h. The resulting immobilized enzyme is hereafter called “immobilisate.”

#### Binding capacity

Binding capacity of the different carriers was determined by removing the enzyme solution after immobilization and measuring its protein concentration according to Bradford (1976). The amount of immobilized protein has been calculated according to Formula 1 with *K* representing the binding capacity in percent. Here, *m* represents the protein concentration of the enzyme solution after immobilization and *β* the protein concentration of the enzyme solution before immobilization.

1 $$K =\left(1 - \left(\frac{m}{\beta }\right)\right) \cdot 100$$

#### Leakage

The leakage of the bound enzyme was determined in two cycles after immobilization. Therefore, 1 mL of 80 mM PPB was added to the loaded carriers. The carriers were incubated at 800 rpm, 4 °C for 2 h. Afterward, the washing solution was removed, and its protein content was determined according to Bradford (1976).

#### Enzyme recycling

Kinetic measurements for immobilized enzymes were conducted for four min as this time reflects the linear part of the saturation curves in photometric assays. For the reaction, 20 µL of enzyme solution have been used for immobilization; *p*NPO was used as a substrate. The mixture was incubated at 30 °C, 800 rpm for 4 min. Two hundred microliters of supernatant was used to measure the absorbance at 405 nm. After each reaction cycle, 1 mL of PPB has been added to the immobilized enzymes. Washing has been conducted for 15 min, 800 rpm at 4 °C with 1 mL of PPB, and the supernatant was removed with a syringe. The activity of each carrier has been documented over 13 or 14 cycles.

### Enzyme characterization

#### Thermofluor assays

For Thermofluor assays, free PCI_Lip or mutants were dissolved in water. Each preparation was composed of 21 µL buffer (including additives), 2 µL of enzyme, and 2 µL 62.5× SYPRO® Orange Protein Gel Stain (Sigma-Aldrich) (see Supplementary Tables S2 and S3 for all buffers and additives tested). For the determination of the melting curves, a real-time PCR-cycler (Applied Biosystems, Darmstadt, Germany) with excitation at 470 nm and detecting emission at 520 nm was used. Samples were conditioned at 15 °C for 5 min. Subsequently, melting curves were measured from 15 to 95 °C applying a temperature gradient of 1 °C ∙ min^−1^.

#### Enzyme stability

The *T*_50_^60^ values of free and immobilized PCI_Lip and its mutants were investigated according to Broel et al. (2022).

#### Storage stability

Storage stability was examined under various buffer conditions. Therefore, free and immobilized PCI_Lip and mutants were stored over a period of 28 days at 4 °C. Activity was measured photometrically after storage (1, 10, and 28 days) using *p*NPO as substrate for WT and *p*NPH as substrate for the mutants.

### Determination of regioselectivity

#### Conversion of triglycerides

Trioctanoate (Sigma-Aldrich) was dissolved in DMSO to a final concentration of 0.12 M as substrate. Nine hundred microliters of enzyme solution, 450 µL of 5 mM HEPES (Carl Roth) buffer pH 8.0, and 450 µL of substrate were incubated at 30 °C, 900 rpm for 30 min and 2 h. For a blank, the enzyme solution was substituted by 80 mM PPB pH 7.0. All conversions were conducted as duplicates.

#### Analysis of hydrolysis products by ^1^H-NMR

After the conversion, the samples were frozen at −80 °C and subsequently lyophilized at −59 °C and 1.5 mbar overnight. The freeze-dried samples were dissolved in 0.8 mL acetone-D_6_ (VWR Chemicals, Darmstadt, Germany) by mixing for 1 min. Samples were transferred to 5 mm nuclear magnetic resonance (NMR) tubes and subjected to ^1^H-NMR analysis using standard instrument software. The measurements were performed on an Advance II 400 MHz NMR spectrometer (Bruker Biospin, Rheinstetten, Germany) at room temperature. To ensure the correct quantification of samples, the zg30 pulse sequence was used for proton NMR spectra acquisition utilizing a 30° pulse angle, 16 scans, and a relaxation delay of 5.1 s. NMR spectra processing and quantification were performed using TopSpin 4.0 (Bruker Biospin). The spectra were referenced to the residual proton peak of acetone-D_6_ at 2.05 ppm.

## Results

### Immobilization of PCI_Lip

**Fig. 1:**
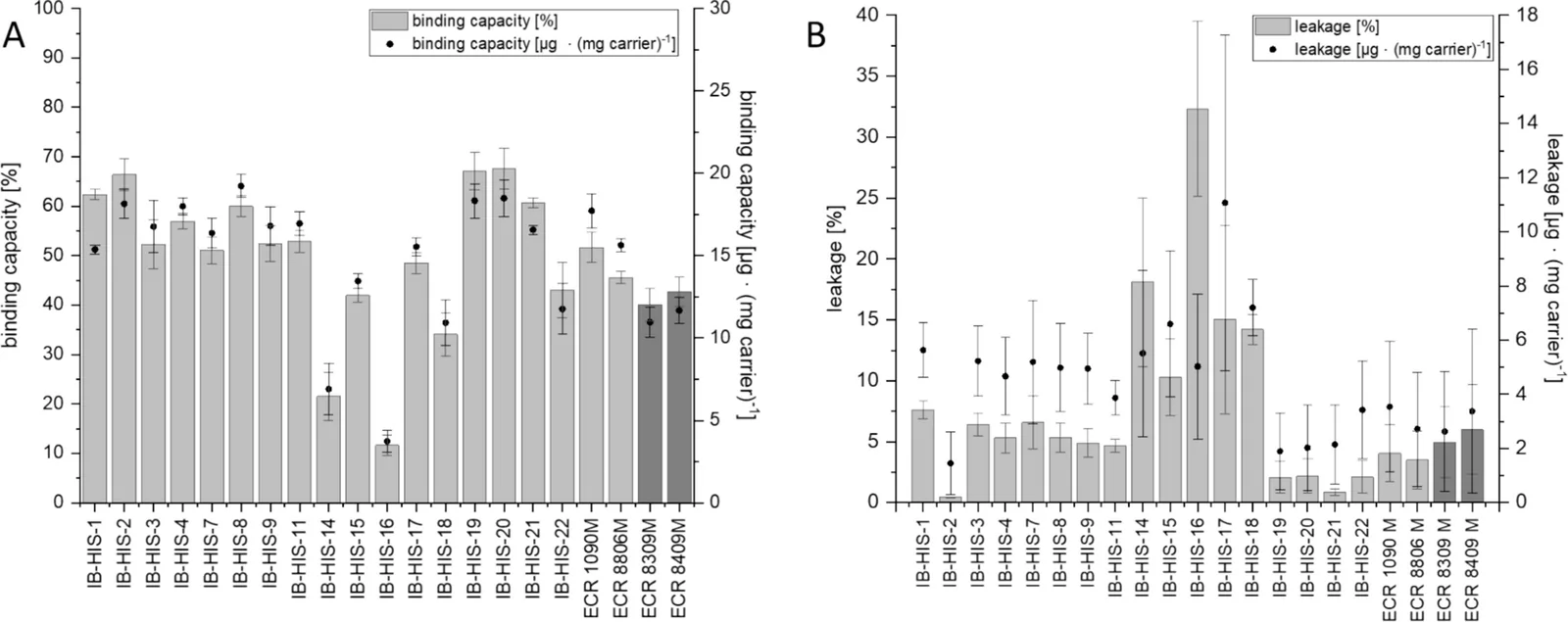
**A** Binding capacities of PCI_Lip on various carriers. The left axis displays the percental binding capacity, while the right axis shows the total binding capacity. Light gray bars represent carrier materials for adsorptive or affinity immobilization, and dark gray bars denote supports for covalent binding. Measurements were conducted in duplicates, with error bars representing the half span. The difference in mean values at significance level of 0.05 was not significant with an *F*-value of 0.3. **B** Leakage of PCI_Lip from different carriers. The left axis displays the percental leakage, and the right axis shows the total leakage. These carriers are distinguished by color: light gray for adsorptive or affinity carrier materials and dark gray for covalent binding carriers. Measurements were conducted in duplicates, with error bars indicating the half span. The difference in mean values at significance level of 0.05 was not significant with an *F*-value of 1.6

**Fig. 2:**
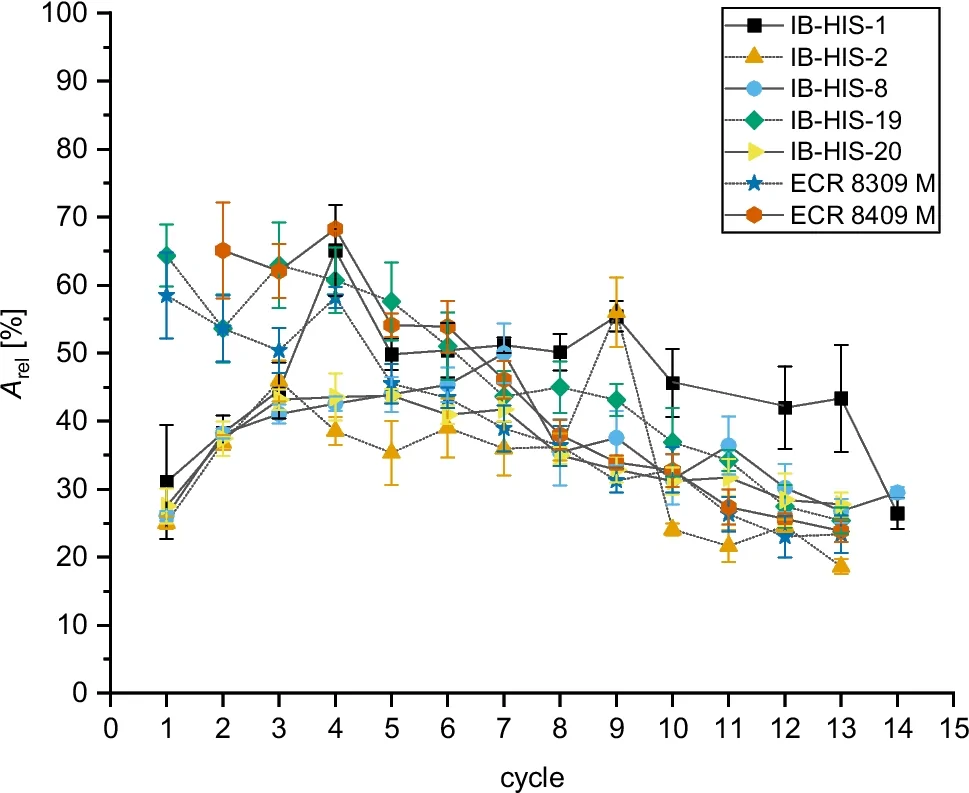
Relative activity of immobilized PCI_Lip on seven selected affinity and covalent carriers compared to the free enzyme over several reaction cycles. Activity was measured photometrically in triplicates using *p*NPO as model substrate. Error bars represent the standard deviation. The difference in mean values at a significance level of 0.05 was significant with an *F*-value of 2.8

PCI_Lip was successfully immobilized to 21 carriers, while 17 used the affinity immobilization His_6_-tag of PCI_Lip. These carriers vary in polarity due to different matrices and functional groups (Supplementary Table S1). Adsorptive immobilization was conducted on two carriers (ECR 1090 M, ECR 8806 M), and covalent immobilization was conducted on another two carriers (ECR 8309 M, ECR 8409 M). The binding capacity and leakage data are given in percent and in total for better comparison (Fig. 1A). Binding capacities vary from 3.7 ± 0.7 µg protein ∙ (mg carrier)^−1^ to 19.2 ± 0.7 µg protein ∙ (mg carrier)^−1^. The carriers IB-HIS-2, 4, 8, 19, and 20 showed the highest binding capacities from 18.1 ± 0.9 µg protein ∙ (mg carrier)^−1^ to 19.2 ± 0.7 µg protein ∙ (mg carrier)^−1^. Generally, polarity did not seem to impact the binding capacity of PCI_Lip to the different carriers, as for the best ones it is indicated from very hydrophobic to very hydrophilic. Adsorptive immobilization led to binding capacities of 17.7 ± 1.0 µg protein∙ (mg carrier)^−1^ for ECR 1090 M and 15.6 ± 0.4 µg protein ∙ (mg carrier)^−1^ for ECR 886 M. Binding capacities of the covalent carriers with 11 ± 1 µg protein ∙ (mg carrier)^−1^ were rather low. To further characterize the aptitude of these 21 different carriers for immobilization of PCI_Lip, the enzyme leakage after two h was determined (Fig. 1B). IB-HIS-2 showed the lowest leakage with less than 1.5 ± 1.2 µg protein ∙ (mg carrier)^−1^. IB-HIS-19, 20, and 21 also scored well in leakage, with 1.9 ± 1.4 µg protein ∙ (mg carrier)^−1^ to 2.1 ± 1.5 µg protein ∙ (mg carrier)^−1^. From the set of 21 carriers, five IB-HIS carriers and the two covalent carriers were chosen to be investigated in a recycling experiment (Fig. 2). The activity of these specific immobilisates was compared to that of free PCI_Lip, which displayed an activity of 565.9 U ∙ L^−1^. IB-HIS-1, 2, 8, 19, and 20 were selected because of their comparatively high binding capacity, all in a similar range between 15.3 and 19.2 µg protein ∙ (mg carrier)^−1^ and rather low leakage. Simultaneously, these support materials cover a broad polarity range from very hydrophobic to very hydrophilic. At the beginning of the recycling experiment, IB-HIS-1, 2, 8, and 20 started with a relative activity of 25 ± 1% to 31 ± 8%. The course of relative activity within these carriers was similar, starting to increase in the first couple of cycles and then decreasing to 18 ± 1% to 29 ± 1%. The initial relative activity of IB-HIS-19 of 65 ± 5% was comparable to the two covalent carriers with 65 ± 7% and 58 ± 6%. These three carriers exhibited an ongoing decrease of relative activity during the cycles, with final relative activities between 23 ± 3% and 25 ± 2%. The relative activity of IB-HIS-2 and 20 increased within the first three cycles to over 40% and then started to decline. For IB-HIS-1, the highest relative activity of 65 ± 7% is reached after four cycles. Next, the relative activity drops to below 50% but remains at this level for the following eight cycles, just dropping to 26 ± 2% in the final cycle. IB-HIS-8 reached the maximal relative activity of 50 ± 4% after seven cycles and continuously decreased afterward. To further investigate immobilized PCI_Lip and its mutants, IB-HIS-19 was selected because of its high binding capacity and low leakage combined with the highest relative activity in several cycles of recycling.

**Fig. 3:**
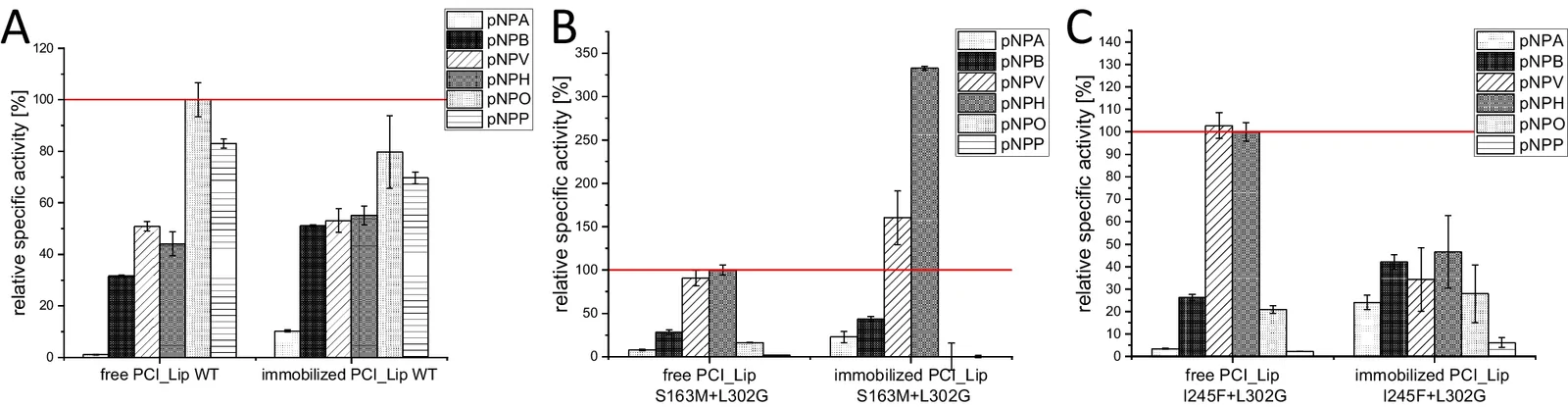
Hydrolysis profiles of PCI_Lip WT and the two mutants (S163M+L302G and I245F+L302G). **A** Relative specific activities of six different *p*NP esters of the free and immobilized PCI_Lip WT compared to the activity of the free WT to *p*NPO (100%, red line). **B** Relative specific activities of six different *p*NP esters of the free and immobilized PCI_Lip S163M+L302G compared to the activity of free S163M+L302G to *p*NPH (100%, red line). **C** Relative specific activities of six different *p*NP esters of the free and immobilized PCI_Lip I245F+L302G compared to the activity of free I245F+L302G to *p*NPH (100%, red line). The difference in mean values at significance level of 0.05 was significant with an *F*-value of 52.0

Next, the hydrolysis profiles of PCI_Lip WT and the two double mutants S163M+L302G and I245F+L302G, which exhibit an altered selectivity towards short- and medium-chain fatty acids (Henrich et al. 2026), immobilized to IB-HIS-19, were examined to see how immobilization affects activity and selectivity (Fig. 3). The activity was normalized to *p*NPO of the free WT, while for the free mutants it was normalized to *p*NPH as they are not active on *p*NPO. The immobilized WT showed slightly higher activity towards short-chain substrates. Activity towards medium- and long-chain substrates was reduced by 10–20%. Immobilization of the WT did not cause major changes in the activity and selectivity of the enzyme (Fig. 3A). As mutants S163M+L302G and I245F+L302G have altered selectivities, *p*NPO is no longer the preferential substrate, but selectivity was shifted more towards short-chain fatty acids. Both mutants are strongly reduced in hydrolytic activity against *p*NPO; therefore, relative specific activity is calculated for *p*NPH. The hydrolysis profile of immobilized S163M+L302G did not change the enzyme selectivity. However, immobilization boosted the activity against *p*NPV and *p*NPH. These substrates were converted with 1.5- or 3-fold increase compared to the free enzyme (Fig. 3B). The immobilization of I245F+L302G affected both activity and selectivity. The free mutants predominantly hydrolyzed *p*NPV and *p*NPH among the tested esters. Immobilization reduced the specific activity for two substrates by more than half of the activity in comparison to the free enzyme. Besides the lower activity, selectivity has been changed to more intensely hydrolyze short-chain fatty acids such as *p*NPA but also long-chain fatty acids such as *p*NPP (Fig. 3C).

### Stabilization of PCI_Lip

**Fig. 4:**
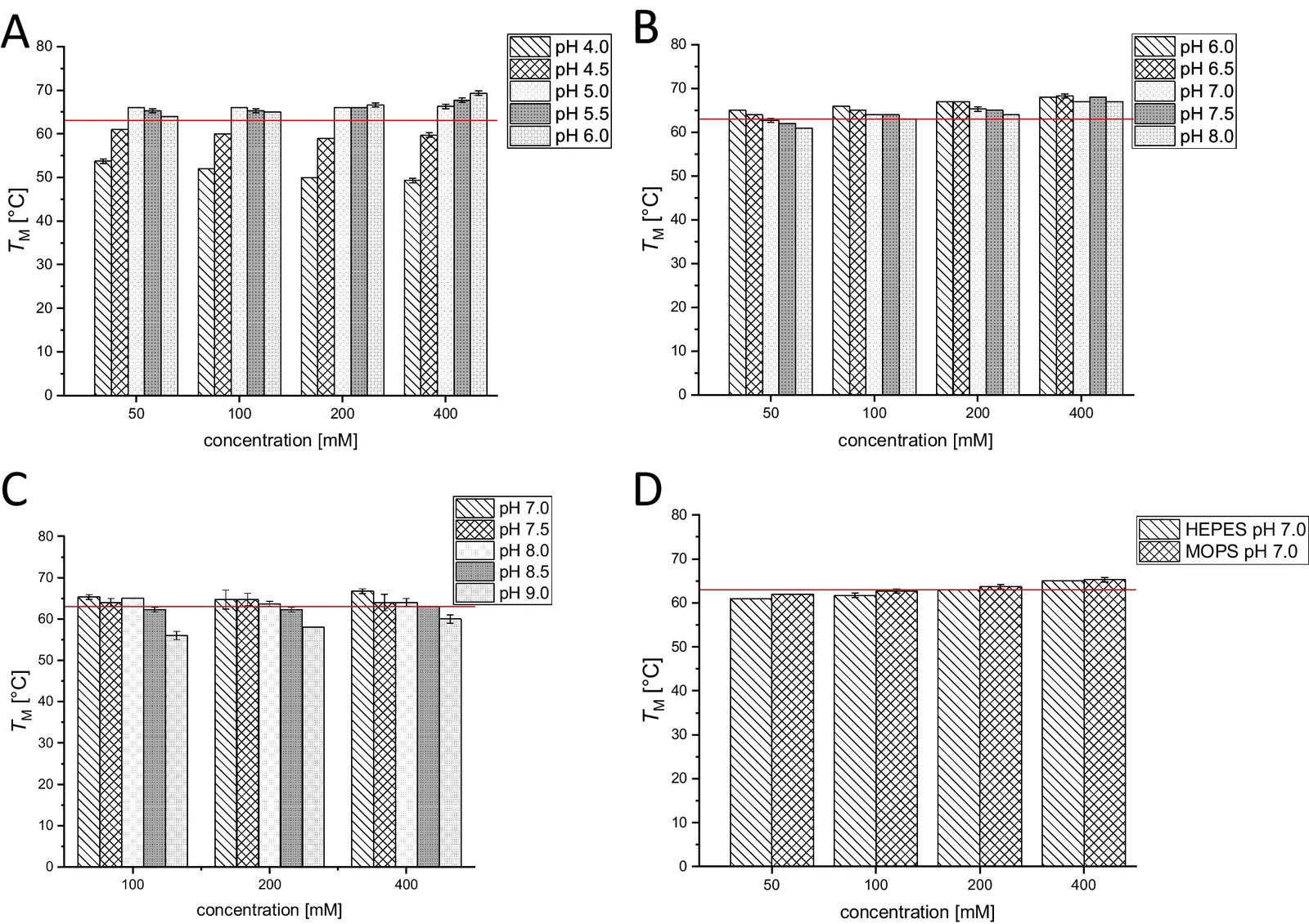
**A** Melting temperature of PCI_Lip WT using various concentrations and pH values of citrate buffer. The red reference line indicates the melting temperature of PCI_Lip WT in water. The difference in mean values at significance level of 0.05 was significant with an *F*-value of 2.8. **B** Melting temperature of PCI_Lip WT using different concentrations and pH values of potassium phosphate buffer. The red reference line shows the melting temperature of PCI_Lip WT in water. The difference in mean values at significance level of 0.05 was not significant with an *F*-value of 1.2. **C** Melting temperature of PCI_Lip WT using different concentrations and pH values of TRIS buffer. The red reference line represents the melting temperature of PCI_Lip WT in water. The difference in mean values at significance level of 0.05 was significant with an *F*-value of 22.3. **D** Melting temperature of PCI_Lip WT using different concentrations of HEPES and MOPS buffers, each at pH 7.0. The red reference line denotes the melting temperature of PCI_Lip WT in water. The difference in mean values at significance level of 0.05 was not significant with an *F*-value of 0.4

**Fig. 5:**
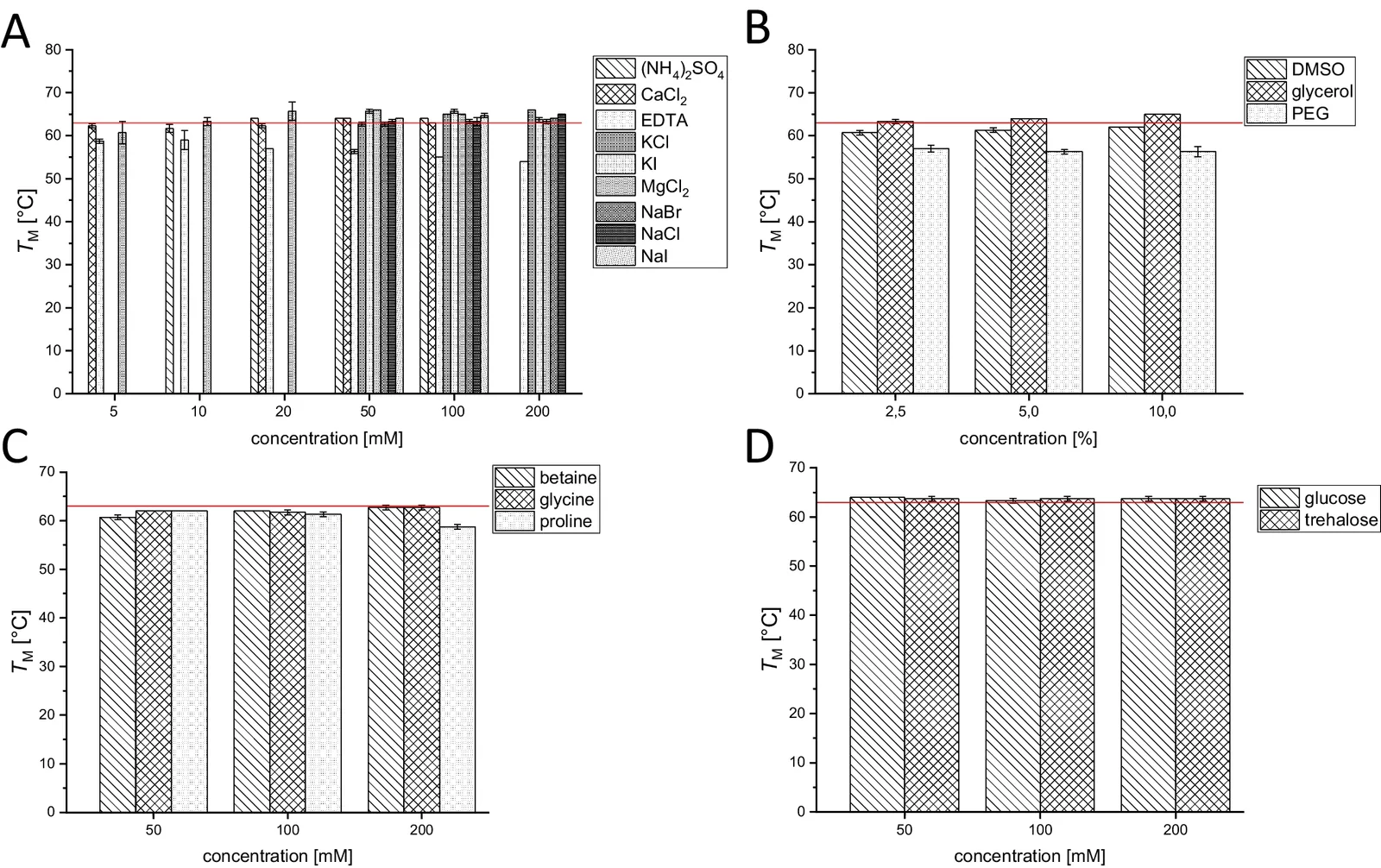
**A** Melting temperature of PCI_Lip WT using various concentrations of selected salts. Different salts were tested at different concentrations according to literature references. The red reference line indicates the melting temperature of PCI_Lip WT in water. The difference in mean values at significance level of 0.05 was significant with an *F*-value of 14.7. **B** Melting temperature of PCI_Lip WT using different concentrations of polyols and DMSO. The red reference line shows the melting temperature of PCI_Lip WT in water. The difference in mean values at significance level of 0.05 was significant with an *F*-value of 100.2. **C** Melting temperature of PCI_Lip WT using different concentrations of amino acids. The red reference line represents the melting temperature of PCI_Lip WT in water. The difference in mean values at significance level of 0.05 was not significant with an *F*-value of 1.2. **D** Melting temperature of PCI_Lip WT using different concentrations of sugars. The red reference line denotes the melting temperature of PCI_Lip WT in water. The difference in mean values at significance level of 0.05 was not significant with an *F*-value of 0.2

**Fig. 6:**
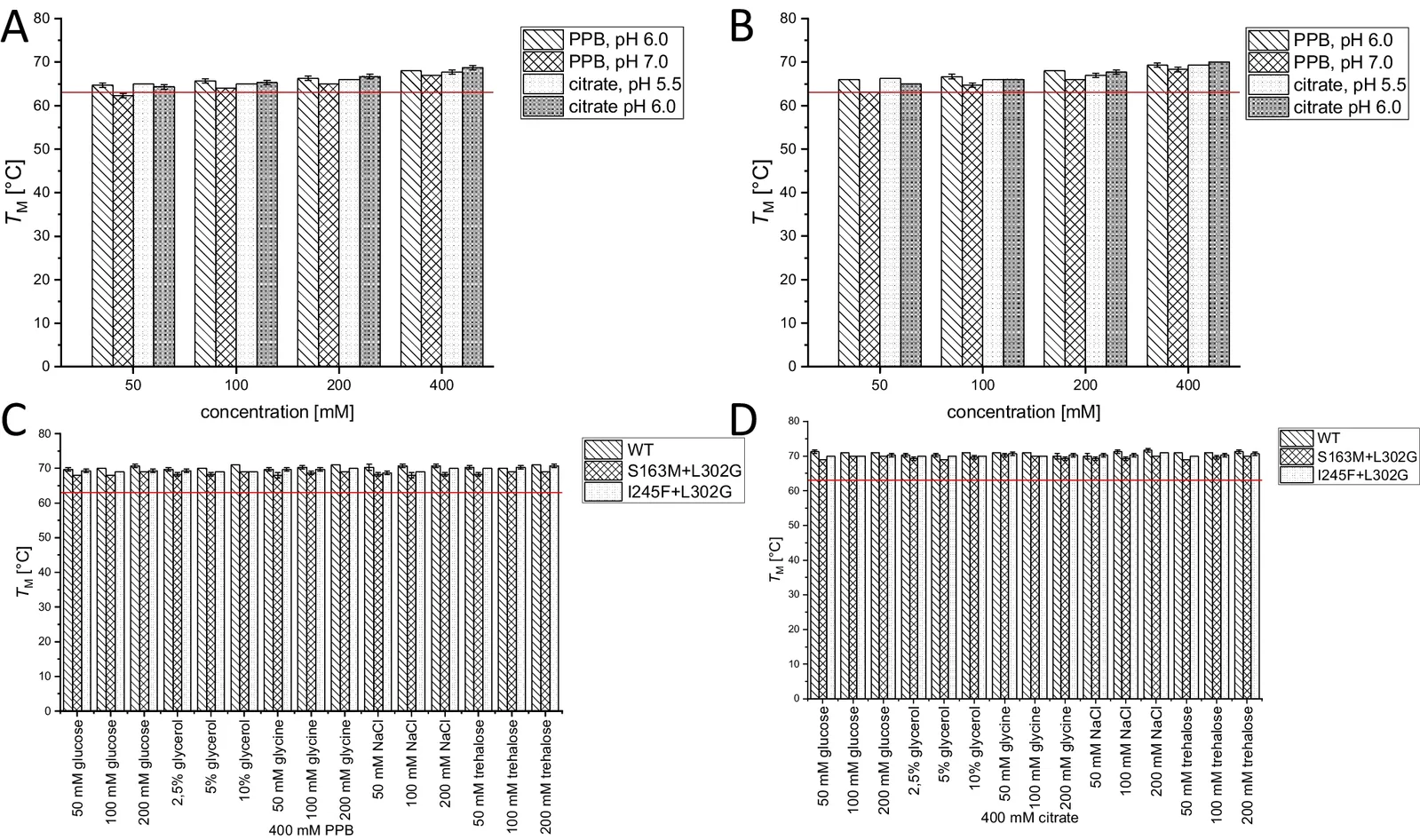
**A** Melting temperature of PCI_Lip S163M+L302G using potassium phosphate buffer (PPB) and citrate buffer at various concentrations and the pH values that performed best in previous measurements with PCI_Lip WT. The difference in mean values at significance level of 0.05 was not significant with an *F*-value of 0.9. **B** Melting temperature of PCI_Lip I245F+L302G using PPB and citrate buffer at different concentrations and the pH values that performed best in previous measurements with PCI_Lip WT. The difference in mean values at significance level of 0.05 was not significant with an *F*-value of 0.9. **C** Melting temperature of PCI_Lip WT and the two double mutants using 400 mM PPB at pH 7.0 and different additives. The difference in mean values at significance level of 0.05 was significant with an F-value of 36.2. **D** Melting temperature of PCI_Lip WT and the two double mutants using 400 mM citrate buffer at pH 6.0 and different additives. The red reference line indicates the melting temperature of PCI_Lip WT in water. The difference in mean values at significance level of 0.05 was significant with an *F*-value of 16.8

**Fig. 7:**
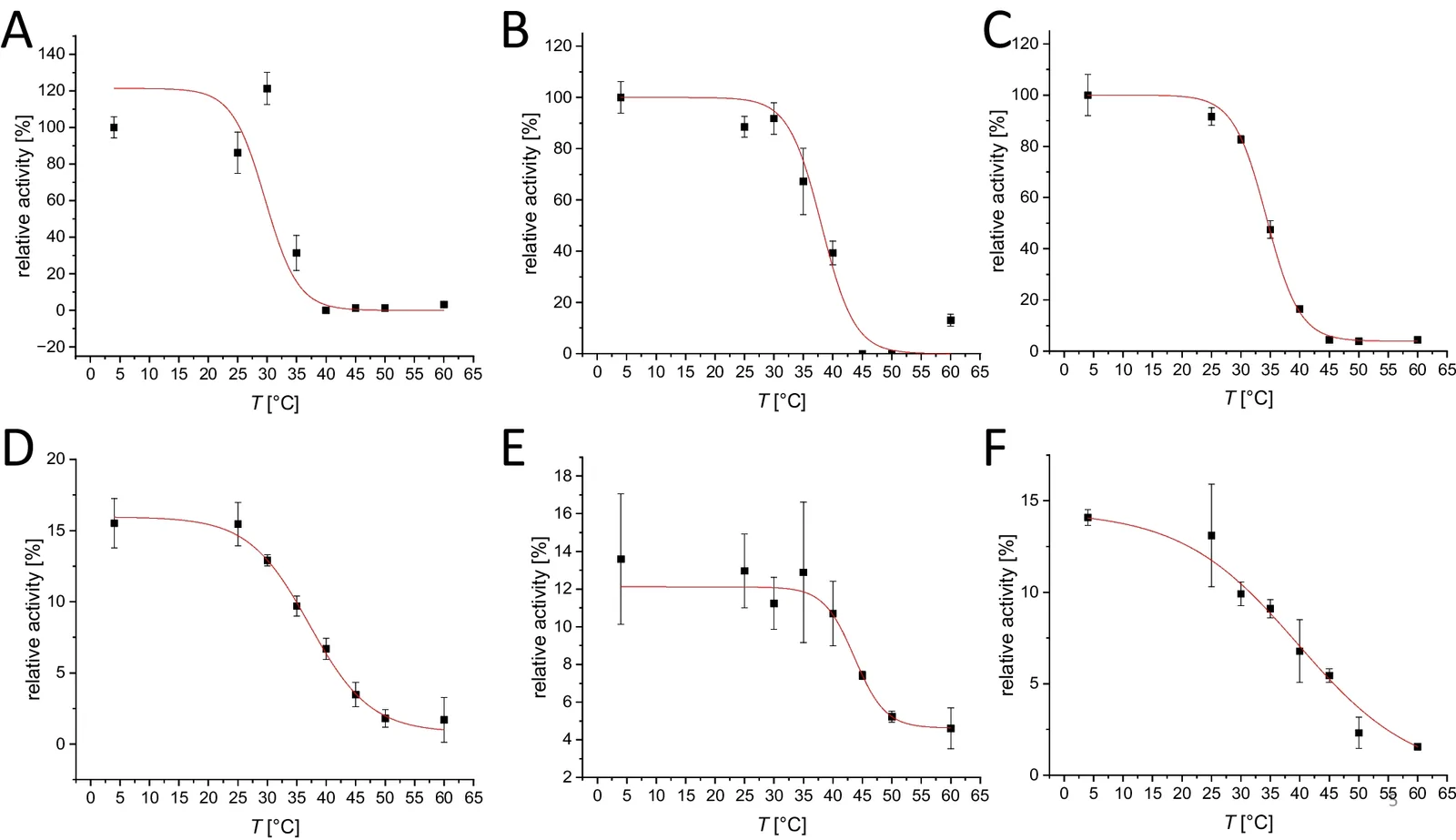
Top row: Activities of free PCI_Lip WT (**A**), S163M+L302G (**B**), and I245F+L302G (**C**) after incubation at different temperatures measured photometrically using *p*NPO for the WT and *p*NPH for the mutants as substrate. All samples were measured in triplicates, error bars represent standard deviation. The activity data were fitted in OriginPro® 2023 using Boltzman fit. The turning point of the curve constitutes the *T*_50_^60^ value of this mutant. Bottom row: Activities compared to the respective free enzyme activity of immobilized PCI_Lip WT (**D**), S163M+L302G (**E**), and I245F+L302G (**F**) after incubation at different temperatures measured photometrically using *p*NPO for WT and *p*NPH for the mutants as substrate. All samples were measured in triplicates, error bars represent standard deviation. The activity data were fitted in OriginPro® 2023 using Boltzman fit. The turning point of the curve constitutes the *T*_50_.^60^ value of this mutant. For confidence intervals and determination coefficients, see Supplementary Table S4

The melting temperature *T*_M_ of PCI_Lip WT in ultrapure water was determined to be 63 ± 0 °C. First, the most suitable buffer, concentration, and pH value for improved enzyme stability were investigated (Fig. 4). The melting temperatures showed that PCI_Lip WT is most stable at slightly acidic to neutral pH values. From pH 5.0 to 8.0 almost all melting points with citrate buffer, PPB and TRIS buffer displayed higher melting temperatures compared to the reference. More acidic and alkaline pH values led to a destabilization of PCI_Lip and *T*_M_ dropped below 60 °C. Within the optimum pH range a higher concentration of the buffer supported the stability of the lipase. This effect was especially visible for HEPES and MOPS buffers. There was no major difference between both buffers. For both buffers, the lowest concentration was destabilizing, but with increasing concentration, *T*_M_ rose above the reference. As a conclusion from the buffer screening, citrate buffer pH 5.5 and 6.0 as well as PPB pH 6.0 and 7.0 each 400 mM were the best for stabilizing PCI_Lip WT increasing *T*_M_ up to 6 °C. Further additives were screened to analyze their effects on enzyme stability (Fig. 5). Most of the screened salts stabilized the enzyme. The stabilizing effect increased with higher concentration for all alkaline chlorides, while it was most prominent for KCl. For MgCl_2_, stability decreased with increasing concentration. The addition of CaCl_2_ did not show major impact on the melting temperature. Higher concentrations of ammonium sulfate enhanced the thermostability at higher concentrations. Potassium iodide stabilized PCI_Lip better than sodium iodide. Among the sodium halogenides, iodide had the highest increase and bromide the least increase of *T*_M_; however, all of them increased the melting temperature with increasing concentration. The effect of increasing stability with concentration was also identified for glycerol. DMSO and polyethylene glycol reduced *T*_M_ of PCI_Lip. Also, the addition of different amino acids lowered *T*_M_. Adding sugars to PCI_Lip increased its thermodynamic stability slightly but no effect of the concentration was observed. Furthermore, the two double mutants S163M+L302G and I245F+L302G were investigated on their thermodynamic stability (Fig. 6A, B. Melting temperatures were determined in the four best buffers identified in the buffer screening for the WT. The mutations S163M+L302G did not affect the thermodynamic stability as melting temperatures are the same as PCI_Lip WT in these buffers. The mutations I245F+L302G had a positive effect on the thermodynamic stability compared to the PCI_Lip WT as *T*_M_ for each buffer composition was increased by 1 ± 0.3 °C. Finally, the thermodynamic stability of PCI_Lip and both double mutants in 400 mM citrate buffer pH 6.0 and 400 mM PPB pH 7.0 with a variety of additives was investigated (Fig. 6C, D). Overall, combining the most stabilizing buffers with favorable additives further increased the stability of PCI_Lip. No negative effects were observed by combining the buffers with the additives and only a few combinations did not change *T*_M_. Within this set, PCI_Lip WT in 400 mM PPB pH 7.0 with 200 mM glycine and 200 mM trehalose respectively showed the highest rise in *T*_M_ with 4 ± 0.5 °C compared to the melting temperature in this buffer. All in all, the highest melting temperatures of 71 ± 0 °C were reached by the WT in 400 mM citrate buffer pH 6.0 with the addition of high concentrations of glucose and trehalose. In summary, the most effective additives to elevate thermodynamic stability were 400 mM citrate buffer pH 6.0 or PPB pH 7.0 combined with 10% glycerol or 200 mM of NaCl, glucose or trehalose. Besides the thermodynamic stability, kinetic stability was examined by monitoring the activity of the lipase after incubation for 60 min at different temperatures. *T*_50_^60^ values were determined for the free and immobilized mutants (Fig. 7). The *T*_50_^60^ values help to evaluate changes in the kinetic stability of an enzyme by representing the temperature where the enzyme activity is reduced by 50% after 1 h of incubation. For the free WT, *T*_50_^60^ is calculated to be 29.7 ± 2.8 °C; for mutant I245F+L302G, *T*_50_^60^ is calculated to be 34.4 ± 0.3 °C; and for S163M+L302G, 38.1 ± 1.2 °C. The immobilized WT had a *T*_50_^60^ value of 37.1 ± 0.7 °C, while for S163M+L302G *T*_50_^60^ is 43.0 ± 0.4 °C and for I245F+L302G it is 39.6 ± 1.6 °C.

**Fig. 8:**
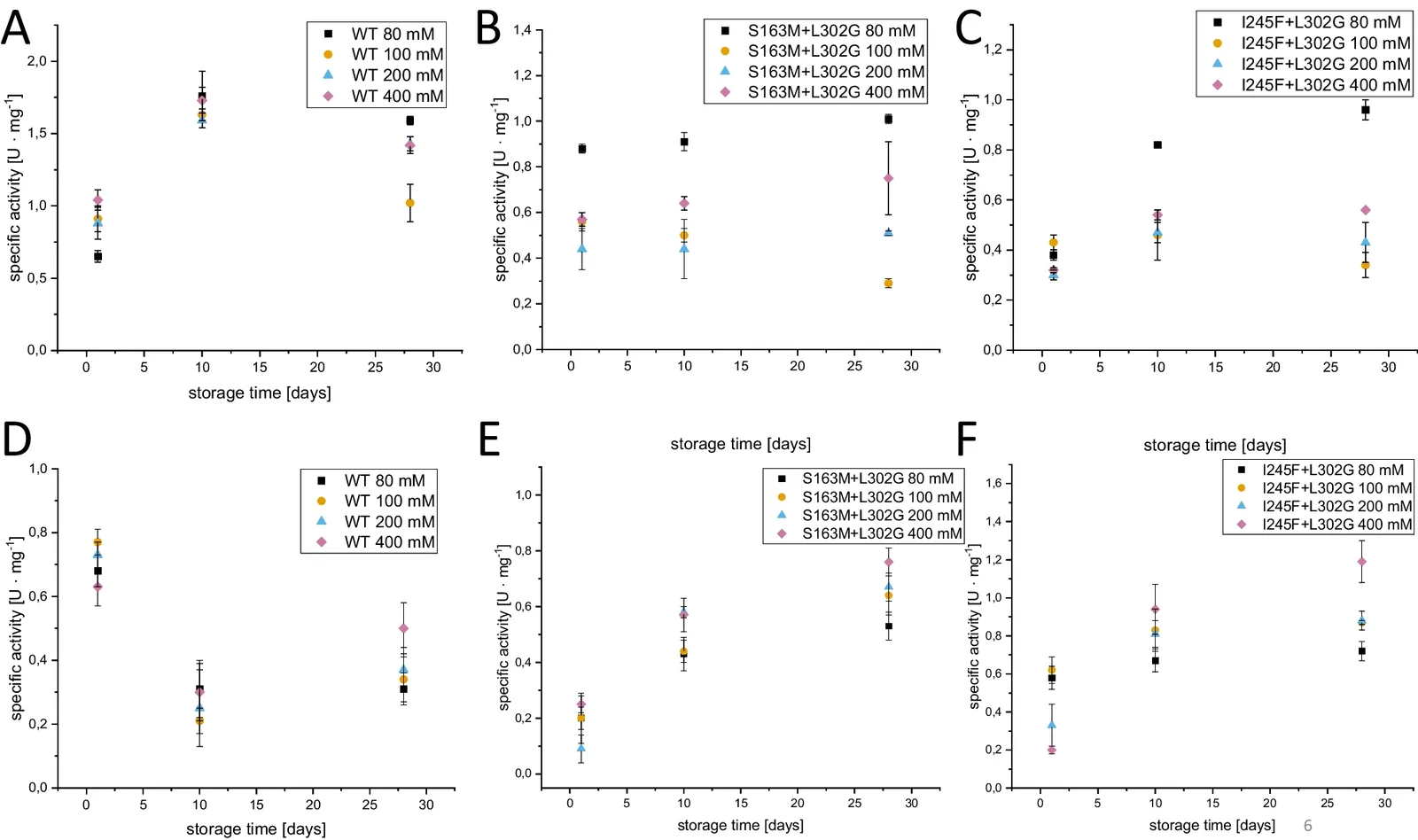
Storage stability of PCI_Lip WT and variants over a period of 28 days in different potassium phosphate buffers (PPB), evaluated by specific activity: **A** PCI_Lip WT, **B** PCI_Lip S163M+L302G, **C** PCI_Lip I245F+L302G, **D** immobilized PCI_Lip WT, **E** immobilized PCI_Lip S163M+L302G, **F** immobilized PCI_Lip I245F+L302G. The difference in mean values at significance level of 0.05 was significant with an *F*-value of 13.8

Further the storage stability of free and immobilized PCI_Lip WT and mutants was investigated over a period of 28 days. Therefore, the activity of the lipase after 1, 10, and 28 days of storage at 4 °C was measured (Fig. 8). The highest specific activities were measured with PCI_Lip WT within the first 10 days stored in PPB of any concentration. The WT activity increased within the first 10 days before a decrease after 28 days was noted. During the storage of S163M+L302G, the activity remained almost the same over 28 days. Within the first 10 days of storage of I254+L302G, activity increased and stayed almost the same up to 28 days. The initial specific activities of immobilized PCI_Lip WT stored in PPB were slightly lower than the activity of the free lipase. The activity of PCI_Lip WT decreased within the first 10 days and then stayed almost similar over the remaining storage time. The specific activity of immobilized PCI_Lip S163M+L302G was similar beyond the different concentrations of storage buffer. In this case, activity increased over the whole storage period. In the case of immobilized PCI_Lip I245F+L302G the initial activity of the lipase stored in 200 and 400 mM PPB is comparable to the initial specific activities of the free mutant. Preparations stored in 80 and 100 mM PPB displayed incipiently a specific activity twice as high as the ones of the free mutant. Within the first 10 days, the activity increased in all preparations. Over the remaining storage time activity stayed the same except for immobilized I245F+L302G stored in 400 mM PPB where it further increased.

### Regioselectivity of PCI_Lip WT

After the conversion of trioctanoate for 30 min and 2 h in aqueous buffer at 30 °C, the hydrolysis products were investigated by ^1^H-NMR analysis (Supplementary Figure S1). The respective compounds and the associated proton shifts are listed in Supplementary Table S5. In the ^1^H-NMR spectra of the hydrolysis mixture, one can distinguish peaks of protons from the glycerol group of the triglyceride (TG) initially added as substrate and peaks that can be assigned to di- and monoglycerides. Evaluation was conducted with the help of NMR spectra of standard glycerides, also reported in the Supplementary Figures S2–S5. The results of the quantification of all glycerides in the ^1^H-NMR spectra of hydrolysis mixtures in duplicate are reported in Supplementary Table S6, along with blank experiments. 1,2-Diglycerides (DG) and 1-monoglycerides (MG) are the most important products of TG hydrolysis already at 30 min, and their amount is even higher at 2 h. In conversion experiments using 1,2- and 1,3-DGs without lipase, an acyl shift was observed for both types of DGs after 30 min in aqueous buffer at 30 °C. As shown in Supplementary Figure S6, 60% of 1,2-DG was transformed to 1,3-DG in 30 min, while only 20% of 1,3-DG was isomerized to 1,2-DG in the same time. This indicates a much stronger acyl shift for 1,2-DGs. The strong acyl shift for 1,2-DGs implied that the initial amounts of 1,2-DGs produced were even higher than those observed in the NMR spectra. Some of it was isomerized directly to 1,2-DGs, indicating a strong preference of the enzyme for the outer positions of TGs. The presence of high quantities of 1-MG also points to this direction, as 1,3-DGs only have outer ester groups that the enzyme hydrolyzed to produce 1-MGs. From these findings, we concluded that PCI_Lip WT displayed a preferential regioselectivity for fatty acids esterified in the outer positions of a TG in the first stage of hydrolysis at the tested conditions.

## Discussion

### Immobilization

#### Binding capacity and leakage of different carriers

Generally, the best immobilization rates were achieved on the IB-HIS carriers with affinity binding of the lipase via a His_6_-tag. However, no conclusion can be drawn concerning the functionalization of the carriers or polarity that promoted binding of PCI_Lip. IB-HIS-1 is classified as very hydrophobic due to the high amount of butyl groups and low amount of Ni-IDA. It displays a similar binding capacity as IB-HIS-20 functionalized with ammonia groups and Ni-NTA, which is classified as very hydrophilic. The particle size reached from 150 to 710 µm, while the supplier offers no information on the pore size. Beyond all tested IB-HIS carriers, the particle size is mostly similar, only IB-HIS-15 with 250–400 µm, IB-HIS-16 with 20–40 µm, and IB-HIS-17 with 100–250 µm deviate from the average of 150–710 µm. Studies on immobilized lipases from *Mucor miehei* and *Rhizopus oryzae* revealed that the particle size of the support has no influence on the binding capacity. The main factor concerning binding capacity was found to be the pore size of the support material, which may also influence the activity of the immobilized enzyme (Gustafsson et al. 2012). The advantage of these supports is immobilization by affinity using the His_6_-tag fused to PCI_Lip to immobilize the enzyme. Compared to other immobilization techniques such as adsorptive or covalent binding methods, affinity immobilization uses highly selective interactions between the support and enzyme. This offers a controlled orientation of the enzyme avoiding inactivation by blocking the active site. Further, the mostly peripheral position of affinity tags reduces conformational changes of the enzyme due to immobilization (Anboo et al. 2022). The relative specific activity and hydrolysis profiles of free and immobilized PCI_Lip WT are very similar, which rules out major conformational changes and supports the minimal impact of this immobilization technique. The polar cellulose matrix of IB-HIS-16 might be one reason for its overall low binding capacity. Mostly hydrophobic support surfaces are described as more suitable for effective immobilization of lipases due to their interfacial activation (Deon et al. 2020). Even though, this affinity binding is described as rather weak (Homaei et al. 2013), for PCI_Lip immobilized on the IB-HIS carriers also other factors seemed to have an impact on the binding strength. Putatively, there might be further non-covalent interactions of the lipase with the surface of the support besides the binding of the His_6_-tag to the Ni^2+^-ion. The hydrophobic, mesoporous polystyrene support ECR 1090 M was marginally better than the hydrophilic amino C_2_-derivatized methacrylate support ECR 8806 M, even though the latter had a much lower surface area and smaller pore size. Adsorption is the most favorable immobilization method for industrial applications because of its low cost and simplicity (Ishak et al. 2025). Though the two supports investigated in this study do not seem to be the optimal supports for PCI_Lip WT. Some studies report an increased specific activity of immobilized lipases, which is attributed to the interfacial activation of the lipase being immobilized in its open conformation (Mokhtar et al. 2020). The low amount of lipase immobilized to the adsorptive carriers may influence specific activity of PCI_Lip positively, as the enzyme carries a putative lid domain responsible for interfacial activation (Broel et al. 2022). Both covalent carriers were used in the recycling experiment with glutaraldehyde as the linker and spacer between enzyme and support. This was reported to have positive effects on the activity of immobilized enzymes. By using a spacer in covalent immobilization, the enzyme remains more flexible and steric hindrance is avoided (Zhang et al. 2013). Further, the activity of PCI_Lip after a harsher immobilization treatment should be investigated. As covalent immobilization is described to have a reduced loss of enzyme (Homaei et al. 2013), it is possible that besides the covalent immobilization of PCI_Lip, some of the enzyme was absorptively attached to the support and was washed off during the cycle.

#### Recycling of immobilized PCI_Lip

During the recycling, seven immobilisates could be divided into two groups. The activity of the first group exhibits a similar development as the activity observed for *Candida antarctica* lipase B (Cal B) immobilized on bagasse for the production of biodiesel (Cui and Di Cai 2018). A minor increase during the first cycles may be due to a rearrangement of the immobilized lipase into a superior conformation or position. This could be observed especially when there was a longer storage period up to the following measurement (Gustafsson et al. 2012). The microenvironment around the immobilized PCI_Lip keeps it stable to retain a relative activity above 60% in the beginning. Accessibility of the active site does not seem to be heavily impacted by immobilization to these support materials. However, the stabilization of the enzyme by immobilization can be caused by increased rigidity of the enzyme, hindering conformational changes during the catalysis (Stepankova et al. 2013). This may cause lower initial activity than the free PCI_Lip. But, immobilization to these support materials seemed to result in smaller conformational changes with little impact on the activity than the other four immobilisates. The second group of immobilized enzymes may face restricted active site accessibility or mass transfer limitations, causing the relatively low activity compared to the free enzyme (Robescu and Bavaro 2025). The increase within the first cycles was probably caused by diffusional limitations of the substrate and product. For the recycling, the activity of immobilized PCI_Lip was measured photometrically, using *p*NPO as substrate. Even though the supernatant has been totally removed after each cycle and the carriers have been washed, a slight yellow coloring of the carriers remained, indicating that at least *p*NP could not be removed completely. The increased activity could be attributed to the potential retention of substrate in the immobilisate matrix from previous cycles and elution in the further cycles. Mass transfer limitations are common constraints in enzyme immobilization. For efficient use of immobilized enzymes, free diffusion of substrate and product is necessary. The mobility of both can be restricted by the functionalization of the carrier surface and the pore size of the support. Diffusional limitations can severely impact enzyme activity by reducing the reaction rate, fortifying substrate inhibition and the formation of pH gradients (Hanefeld et al. 2009; Zdarta et al. 2018). In the case of these four IB-HIS carriers, the pore size does not seem to be suitable for the immobilization of PCI_Lip, as the product was held back. Generally, a smaller pore size leads to a larger surface area, which can increase enzyme loading, but it can inhibit mass transfer. Therefore, the ideal support material has to be evaluated for every enzyme immobilization individually (Chen et al. 2019). Selective individual increases in relative activity during the recycling can be described through longer storage periods overnight between the cycles. Storage was conducted in the standard buffer, which seemed to regenerate inactivated, immobilized lipase. These increases in activity are probably assigned to a combination of restricted mass transfer and enzyme regeneration. Chen and Wu describe the regeneration of an immobilized lipase in biodiesel production. The washing removed substrate molecules that stuck to the immobilized enzyme, causing its inactivation (Chen and Wu 2003). In our case, longer storage may have removed perturbing molecules from PCI_Lip. After the recycling experiment, IB-HIS-19 was chosen as the best carrier for further investigations of the immobilized PCI_Lip. The affinity binding of PCI_Lip offered a simple way of immobilization where no hazardous chemicals were needed. This carrier had already displayed a high binding capacity in combination with low leakage. In the recycling experiments, it stood out with high initial activity, which was kept up for five cycles with a slow decline afterward.

#### Characterization of immobilized PCI_Lip

For PCI_Lip WT the advantage of affinity immobilization becomes clear. The immobilization on the peripheral affinity tag of PCI_Lip did not cause severe conformational changes or hinder the flexibility of the enzyme. The affinity tag of PCI_Lip ensures controlled orientation of the enzyme without the risk of activity loss caused by blocking the active site. The attachment of the enzyme on the His_6_-tag causes only minor conformational changes as opposed to other immobilization techniques. Either adsorption or covalent binding always affects the surface of the enzyme, sometimes even at multiple points. This enhances the stability of the enzyme by making it more rigid. However, the increased rigidity may cause reduction or even loss of activity (Anboo et al. 2022). Besides acting as the chelating agent of the divalent nickel ion the nitrilotriacetic acid is a spacer between the carrier surface and the enzyme. This enlarges the orientation space of the enzyme further reducing conformational restrictions. Also the higher flexibility of the enzyme leads to a stronger binding (Alács et al. 2024). The slight decrease of activity of immobilized PCI_Lip WT is caused by the interaction of the lipase with the carrier surface. Besides the attachment via the His_6_-tag parts of the enzyme surface can interact with functional groups of the carrier surface by means of hydrophobic and van-der-Waals interactions as well as hydrogen bonds (Alács et al. 2024; Tzialla et al. 2009). The additional adsorptive interactions further stabilized the immobilized PCI_Lip. This goes along with the increased *T*_50_^60^ values of the immobilized enzyme compared to the free one. However, the increased stability engendered a slight reduction of the activity. Further hydrolysis profiles of the two immobilized double mutants S163M+L302G and I245F+L302G have been compared to their free versions. These mutants have been created to shift the selectivity of PCI_Lip to an increased hydrolysis of short- to medium-chain fatty acids and also reduce the hydrolysis of long-chain fatty acids. Therefore, amino acids mainly in the binding pocket of PCI_Lip have been substituted (Henrich et al. 2026). For S163M+L302G the activity for its preferential substrates *p*NPV and *p*NPH is strongly increased. Several studies describe an increased activity of lipases when immobilized (Cui and Di Cai 2018; Deon et al. 2020; Dong et al. 2021; Tzialla et al. 2009; Zhao et al. 2008). This is often explained by the immobilization of the lipases in their most active state. Most lipases including PCI_Lip share the common feature of a lid domain that covers the active site and opens up, when in contact to a hydrophobic interphase. Especially when using adsorptive immobilization on hydrophobic supports the lipase can be immobilized in its open conformation showing the highest activity. The permanent open conformation of a lipase enables free access of the substrate to the active site and causes considerably higher activity often called hyperactivation (Blanco et al. 2004; Deon et al. 2020). Even though the amino acid exchanges of S163M+L302G are located near and inside the substrate channel, they seem to affect the overall conformation of PCI_Lip. The implementation of methionine and glycine may have changed the conformation of the lipase. This might be in a way that adsorptive interactions are enhanced that favor the open conformation and cause hyperactivation. As mutation L302G is present in both investigated mutants, the hyperactivation seems to be associated with mutation S163M. This one is positioned a little aside the substrate channel and has previously been shown to alter the chain length selectivity towards short- to medium-chain fatty acids (Henrich et al. 2026). The mutation is located further away from the active site, near the substrate channel and the lid domain. It may therefore influence the conformation of the lid domain without noticing beforehand but refining the mutants’ properties when immobilized. The free form of PCI_Lip mutant I245F+L302G is most active against *p*NPV and *p*NPH, but already exhibits reduced activity compared to the WT (Henrich et al. 2026). Immobilization of this mutant reduced its activity by almost half and simultaneously shifted its selectivity. Both substitutions are located in the substrate channel but replacing isoleucine with phenylalanine and leucine with glycine seems to impact on the overall enzyme conformation, leading to a diminished enzyme activity when immobilized. The combination of amino acid substitutions and immobilization lead to a reduced activity in this case. These minor changes can also impact enzyme selectivity. Several factors have been described to putatively influence selectivity when an enzyme is immobilized. The outcome is hard to predict and has to be tested for each enzyme/carrier combination individually. Potential reasons for an alters selectivity in a specific enzyme/support combination may be diffusional limitations, the micro-environment, structural modifications, or a changed rigidity (Rodrigues et al. 2013). In this case, the substitution of isoleucine to the bulkier phenylalanine in combination with the immobilization may downgrade the free diffusion of the substrate molecules into the active site leading to the reduction of activity. A similar change of selectivity has been observed for a lipase from *R. miehei* when immobilized to a resin. This immobilized lipase became more active on short-chain fatty acids as well (Lee and Parkin 2001). The examination of the hydrolysis profiles of PCI_Lip WT and two double mutants underlined that every enzyme/support combination accounts for different results. Immobilization of WT to HB-HIS-19 displayed only minor changes in activity, while immobilization of S163M+L302G showed a hyperactivation and I245F+L302G exhibited reduced activity combined with altered selectivity.

Among other aspects, the aim of this study was to improve the stability of PCI_Lip. Therefore, thermodynamic stability and kinetic stability of the lipase have been investigated. Thermodynamic stability refers to the enzyme conformation and its tendency to denature, while kinetic stability deals with irreversible inactivation and the loss of activity (Iyer and Ananthanarayan 2008). The introduction of two other amino acids in the free double mutants led to an increase in the *T*_50_^60^ value compared to the WT with a *T*_50_^60^ value of 29.6 ± 2.8 °C. Exchanging leucine for glycine at position 302 raised the semi-inactivation temperature for 1.5 °C (Broel et al. 2022). Both double mutants carry this mutation, which has been shown to have a positive effect on the *T*_50_^60^ value. The increase of kinetic stability in the free double mutants compared to the WT is likely a side effect of mutating selected amino acids in or near the substrate channel. Xie et al. have chosen a similar approach to stabilize Cal B. They chose against established approaches that target increased structural rigidity, restricted conformational flexibility or boost the interactions of unstable domains. Since the active site of an enzyme is a rather fragile part, nevertheless vital to catalytic activity, they aimed to stabilize this part of the enzyme by exchanging amino acids near the active site. This strategy led to a mutant with a semi-inactivation temperature 12 °C above that of the WT in the Cal B (Xie et al. 2014). The mutations in the substrate channel stabilized the active site of PCI_Lip, which led to an increased kinetic stability of the free double mutants. The combination of amino acid exchanges seemed to have increased the semi-inactivation temperature even more. Similar effects have been observed in a creatinase, where the combination of mutations led to an increase in *T*_50_ (Bian et al. 2024). Immobilization of PCI_Lip further enhanced the kinetic stability of the enzyme. The semi-inactivation temperature of the WT and both mutants was increased by about 6–7 °C through immobilization. As the increase is similar in all three variants it can be attributed to immobilization, and the individual differences have no major impact here. The enhanced thermal stability was a result of the rigidification of PCI_Lip through immobilization. A more rigid enzyme can resist the denaturing effects of higher temperatures better than the free enzyme with high flexibility (Khan 2021). Our findings go along with literature data showing that immobilization of lipases leads to an enhanced thermostability of the respective enzymes (Wilson et al. 2006; Xie et al. 2014; Costantini and Califano 2021). The enhanced stability of PCI_Lip through immobilization that simultaneously keeps its activity offers the chance for industrial applications. Immobilized PCI_Lip could be implemented in the biotechnological production of biodiesel. Therefore, the current findings could be combined with previous findings, showing that chain length selectivity of PCI_Lip can be altered by protein engineering (Henrich et al. 2026). This can offer a chance to find a variant suitable to hydrolysis of long-chain fatty acids with improved stability when immobilized to a support material such as IB-HIS-19.

### Thermodynamic stability of PCI_Lip and its mutants

Thermofluor assays provide the possibility to examine the structural stability of a protein and the influence of its environment. While for the kinetic stability the hydrolytic activity of PCI_Lip was monitored, the thermodynamic stability monitored the structural denaturation. The melting temperature observed in the Thermofluor assays refers to the temperature at which half of the enzyme is denatured (Iyer and Ananthanarayan 2008). The comparison of kinetic and thermodynamic stability of PCI_Lip displays that the enzyme structures that are essential for the hydrolysis unfolded before the whole enzyme scaffold denatured. Compared to Cal B with *T*_M_ of 54.5 °C, which is often used for industrial purposes, PCI_Lip offers a higher thermostability (Kim et al. 2010). The elevated *T*_M_ brings advantages for putative industrial applications. In industrial processes, higher temperatures are used as reaction rates are increased and substrate solubility is better. In this case, lipases from thermophile organisms offer much higher thermostability; for example, a lipase from *Thermomyces lanuginosus* has a *T*_M_ of 93 °C (Xiang et al. 2023). However, microbial lipases mainly catalyze the hydrolysis of long-chain fatty acids (Chandra et al. 2020). The advantage of PCI_Lip and especially the two double mutants is their selectivity for the hydrolysis of medium-chain fatty acids and its mutants offer a selectivity for short-chain fatty acids (Wang et al. 2025; Henrich et al. 2026). The Thermofluor method applied in this study offered the chance to improve the thermostability of this lipase with special selectivity for short- to medium-chain fatty acids to meet the requirements for an industrial application. An increase in *T*_M_ with higher buffer concentration has also been observed for other proteins. Higher buffer strength leads to a higher concentration of ions in the hydration shell of the enzyme and increases water activity on the protein surface. Both give a better structural order and reduce conformational flexibility, resulting in a more thermostable enzyme (Boivin et al. 2013; Kalisz et al. 1997). The chelating agent EDTA can be added to an enzyme solution in order to avoid oxidation of SH-moieties (Boivin et al. 2013). For PCI_Lip, the addition of EDTA did not help to stabilize the enzyme. The addition of salts is a common tool to elevate enzyme stability. Generally, the stabilizing effect of neutral salts is ascribed to ions binding to charged groups on the protein surface and a competition for solubilization in water. Both enhance the hydrophobic interactions of the protein, boosting its stability. While some ions with chaotropic effects rather destabilize enzymes, ions with kosmotropic effects support stabilization. The effects of each ion on enzyme stability are described by the Hofmeister series (Silva et al. 2018). In this study, chloride, bromide, and iodide as anions were tested on the stability of PCI_Lip. Bromide has a less stabilizing effect on the lipase than chloride, but iodide has a more stabilizing effect. Within the Hofmeister series, chloride is claimed to have no effect on the water structure; bromide is described as an anion with slightly chaotropic effect (Lavelle and Fresco 2003). This chaotropic influence of bromide in comparison to chloride is displayed by PCI_Lip with a lower *T*_M_. According to the Hofmeister series, iodide should be more destabilizing. At this point, the series is not in accordance with our results. By screening the three most common sodium halogenides, iodide displayed the highest increase in *T*_M_. In the case of Hofmeister series for cations, larger ones with lower charge are considered to salt out proteins, while smaller ones with a higher charge, salt proteins (Kherb et al. 2012). According to the Hofmeister series, in this study, Mg^2+^ should stabilize the protein best and K^+^ least good. However, the melting temperatures of PCI_Lip WT were highest with the highest concentration of KCl. With increasing concentration of Mg^2+^, *T*_M_ decreased. Calcium, as another divalent ion, barely affected the stability of PCI_Lip even though it is in similar position as magnesium in the Hofmeister series. This discordance also goes along with the results of magnesium. Ammonium sulfate combines two ions that are supposed to have a positive impact on the enzyme stability (Silva et al. 2018). At this point, it is to mention that the Hofmeister series acts as a general guideline with some conformity to stabilizing ions for PCI_Lip. Effects of ions on enzyme stability are highly individual. This is reflected by the deviance from this theoretical series in our data. For PCI_Lip, an increase in *T*_M_ appears at higher concentrations, but other salts at the respective concentrations enhance stability even more. Polyols in general are known to stabilize proteins. Even though the mechanism behind this effect has not yet been fully elucidated, it is traced back to the promotion of hydrophobic interactions within the enzymes, making them more rigid, which increases thermostability (Balcão and Vila 2015). The addition of glucose, trehalose, and glycerol as polyols increased the thermostability of PCI_Lip. The highest increase in *T*_M_ was observed with the addition of 10% glycerol by 2 °C. Research is ambivalent about the effects of DMSO on protein structures and stability. On the one hand, it is described to have positive effects on protein stability, being a popular and widely used cosolvent in bioassays. On the other hand, in some studies, its destabilizing effect on proteins even at low level concentrations is shown (Cubrilovic and Zenobi 2013; Landreh et al. 2014). PCI_Lip seems to be another example for DMSO having destabilizing effects on the enzyme. Still, this does not mean that DMSO generally affects protein stability in a negative way, as Chan et al. found that the impact of DMSO on enzyme stability is highly protein-dependent. In this case, the individual interactions with charged states and electrostatic repulsions caused destabilization of avidin at 4% DMSO but boosted the stability of a bacterial cytochrome at a concentration of 40% (Chan et al. 2017). PEG had a destabilizing effect on PCI_Lip while high molecular weight, PEG 20k, was used. Most literature describes PEG as crowding agents with positive effects on stability. However, recently, some studies showcased some negative effects. It has become clear that, similar to DMSO, effects are protein-dependent and also the size of PEG matters. Conformational changes caused by high molecular PEG have been proven in a tRNA-synthetase from *E. coli* (Liebau et al. 2024). As high molecular PEG has an amphiphilic character, this might affect hydrophobic parts of the lipase causing destabilization and eventually a lower *T*_M_. Some amino acids have been reported to inhibit protein aggregation and support renaturation (Boivin et al. 2013). Therefore, the impact on *T*_M_ of PCI_Lip by adding betaine, glycine, and proline respectively has been investigated. In this case, none of the amino acids had a positive impact on thermal stability. Even though the improvement of thermostability has not been the initial aim of the protein engineering approach, we decided to investigate the two mutants with the best changes in the hydrolysis profiles in this study. These investigations gave deeper insights in the stability of these mutants for putative industrial applications of a microbial lipase highly selective for short- to medium-chain fatty acids. We suggest that the mutation I245F is causing enhanced stability as the mutation L302G appears in both of the tested mutants. Even though the enhancement of stability was not the initial intention of the protein engineering, the results are in line with other studies aiming to improve thermostability by the exchange of amino acids. The mechanisms underlying the stabilization of an enzyme by the introduction of other amino acids are versatile. In this case, it seems likely that the increased stability could be attributed to the formation of new π-π-interactions with F165 stabilizing this rather flexible region (Bai et al. 2020; Bian et al. 2024). This mutation is located in the substrate channel. Xie and co-workers followed a similar strategy to improve the kinetic stability of Cal B by mutating amino acids in flexible regions near the active site. This increased the rigidity of the enzyme in an area vital for its activity, increasing its half-life time 13-fold (Xie et al. 2014). Finally, the thermostability of PCI_Lip WT and both double mutants was investigated using the best buffer systems with the best additives. The results show that the improvement of the composition of the enzyme solution is a good tool to improve the properties of an enzyme for a potential industrial application. Glycerol, glycine, and trehalose were most effective in increasing *T*_M_ in comparison to the pure buffer. All in all, citrate buffer with glucose, glycerol, glycine, NaCl, and trehalose could increase *T*_M_ to 71 ± 0 °C and PPB with glycerol, glycine, and trehalose reached the same *T*_M_. The improvement of thermodynamic stability of PCI_Lip by selection of appropriate buffer systems gives rise to a broad use of this lipase in biotechnological processes. The high stability makes the lipase a good candidate for a biocatalyst in the production of chemical or pharmaceutical compound, as these reactions are often conducted under elevated temperatures.

### Storage stability of free and immobilized PCI_Lip

An increase of activity of free PCI_Lip WT within the first days after purification has been observed beforehand. We assume that this is attributed to an ongoing refolding of the enzyme during the first days of storage as heterologous expression also leads to the formation of inclusion bodies besides some amount of soluble lipase. Spontaneous refolding is also known for other proteins like different fibronectin domains (Shah et al. 2017). A decrease of activity over a long storage period is commonly observed for various enzymes. Another factor is the storage temperature that can affect stability over a longer storage period. However, over the course of 28 days, several denaturing effects such as pH changes, proteolytic activity, and microbial contamination can lead to enzyme denaturation (Dos Santos et al. 2018). For S163M+L302G, 80 mM PPB seemed to be the most appropriate buffer showing considerably higher activities than the other concentrations over the whole storage time. For this mutant, the use of PPB in appropriate concentration can help to stabilize the enzyme over a longer storage time even supporting refolding which results in an increase in activity. Over the 28-day storage period, the concentration of the buffer has the most impact on the enzyme activity of I245F+L302G. The activity of the preparation in 80 mM PPB increased further indicating that this buffer further favors refolding. Overall, this experiment showed that free PCI_Lip exhibits good storage properties when the most suitable buffer concentration is used. Mostly, the use of 80 mM PPB led to the highest activities of the mutants during storage. Higher stability in the presence of low phosphate buffer concentration has been reported for fibroblast protein before. Here it is assumed that substrate effects are most important in the interaction of enzyme and substrate (Ugwu and Apte 2004; Min Won et al. 1998). However, the application of 100 mM PPB appears to be no good choice for the activity and storage stability of free PCI_Lip. These findings go along with the results reported by Kornecki et al (2020). They showed that 100 mM phosphate buffer pH 7.0 had a negative effect on the stability of different lipases. Also, they were able to show that this effect changes with the use of different buffers and pH values. They assume that the reason is within the lipase structure and not the ionization degree of the phosphate (Kornecki et al. 2020). In accordance with these previous results, we could show that the concentration of the phosphate buffer also influences lipase stability under the given conditions. Generally, this experiment underlines the importance of screening for the appropriate buffer concentration as this affects enzyme activity and stability. The initial activity of immobilized PCI_Lip WT is a little lower than that of the free lipase, which is in accordance with the results of the comparison of the hydrolysis profiles. Within the first 10 days of storage, the activity of all immobilisates decreased strongly, yet it remained at this level for the residual storage time. The comparison of specific activity of immobilisates and free WT suggested that it would be best to store PCI_Lip as free enzyme, which increased activity. Immobilization should be performed immediately before the use of the immobilisates. In the case of the mutants, immobilization supported storage stability, even leading to an increase of activity. In both cases, activity further increased for any buffer concentration, while the activity of the free enzymes remained the same or even decreased. The improved storage stability by immobilization has been reported by several researchers before. Compared to the free enzyme a catalase immobilized on a chitosan/ZnO support the decrease in activity over a period of two month was much lower than for the free enzyme (El-Shishtawy et al. 2021). Al Angari et al. demonstrated that a *Candida rugosa* lipase lost its activity after 30 days of storage while immobilization helped to keep 80% of the initial activity after 60 days of storage (Al Angari et al. 2023). Restricted flexibility and higher rigidity due to structural modifications reducing the effects of denaturation caused by immobilization are discussed as the reasons for the improved storage stability (Atiroğlu 2020). In the case of PCI_Lip, we tried to keep up a higher degree of flexibility by choosing for affinity immobilization via the His_6_-tag. Still, this type of immobilization seemed to affect the conformation of the enzyme in such manner that it improved storage stability, being less prone to denaturation. Both immobilized double mutants displayed an increasing activity over the whole storage period in all buffer concentrations. We assume that the refolding process described for the free enzyme before is expanded over a longer time in the immobilized variants. Probably the refolding of free PCI_Lip can take place faster, which is reflected in an activity increase within the first 10 days as there is no restriction of flexibility. Immobilization diminished the conformational flexibility and, therefore, also the chance to refold. As immobilization prevents the enzyme from denaturation and causes a slower refolding process, an increase in activity has been observed over the whole storage period.

### Investigation of regioselectivity

Substrate selectivity is a key factor in the application of lipases, especially in the industrial field. Substrate selectivity of these hydrolases can be further distinguished into typoselectivity, stereoselectivity, and regioselectivity. The typoselectivity of PCI_Lip, which covers the preference of the enzyme for a certain type of fatty acid, has been investigated in detail beforehand (Park and Park 2022). In several studies, PCI_Lip has been found to be most active on medium- to long-chain fatty acids, especially capric and palmitic acids. Also, the typoselectivity has been adapted towards short- to medium-chain fatty acids via protein engineering (Broel et al. 2022; Sowa et al. 2022; Wang et al. 2025). For an industrial application, knowledge about the regioselectivity is essential to achieve the desired products. Most TG consist of different fatty acids esterified to the three hydroxy groups of the glycerol backbone. Depending on the source of the TG, fatty acids of different chain length are esterified at defined positions (*sn* 1–3) of the glyceryl moiety. As there are differences in the fatty acids esterified in the *sn* 1 and 3 position compared to the *sn* 2 position, regioselectivity of a lipase is an important factor considering the substrate and desired products. In milk fats, for example, short-chain fatty acids are predominantly esterified in the outer positions of a TG. Differently, in palm oil, palmitic acid is mainly esterified in these positions. Fatty acid distribution is a very important topic when it comes to human breast milk. Here, it is key to the nutritional value for the baby, but still most infant formulars show considerable differences to breast milk in that aspect (Qi et al. 2018; Wei et al. 2019; Yener et al. 2021). Within the group of lipases, some almost exclusively hydrolyze fatty acids at the outer positions, *sn* 1 and 3, producing 1,2- or 2,3-DGs. The other part of lipases is summarized under the term non-regioselective lipases hydrolyzing fatty acids at any position of the TG (Lee et al. 2024). In this study, we choose to analyze the products of the conversions of homogeneous TGs by ^1^H-NMR. By using a homogeneous TG, possible influences caused by a preferred hydrolysis of a certain fatty acid are avoided (Rodrigues et al. 2021). The evaluation of the resulting spectra suggests that PCI_Lip WT is *sn* 1,3-regioselective lipase. Besides the peaks assigned to the remaining substrate, the biggest peaks in the spectra could be assigned to 1,2-DG, indicating that these are the main products of the first stage of hydrolysis. Besides 1,2-DG, there also were peaks that were assigned to 1,3-DG. In additional experiments, under the same conditions but without lipase, especially 1,2-DG showed acyl migration. Acyl migration is a thermodynamically driven process describing the migration of fatty acids in a di- or monoglyceride (MG) to another position at the glycerol backbone (Da Silva et al. 2012). Considering the quantitative NMR results of glyceride distribution in the hydrolysis experiments reported in Supplementary Table S6 as well as the results from the migration experiments, we concluded that PCI_Lip is 1,3-regioselective. The small amounts of 1,3-DG probably result from acyl migration. Similar findings concerning acyl migration in diglycerides have been identified when the regioselectivity of immobilized lipozyme RM IM was investigated (Meng et al. 2014). The setup of our experiment combines some factors promoting acyl migration. Acyl migration is a thermodynamically driven process being favored by elevated temperatures. Depending on the reaction conditions, previous studies showed that a reaction equilibrium can be reached within 1 to 2 h (Kodali et al. 1990; Yang et al. 2005). Further, the deployed substrate trioctanoate is reported to enhance acyl migration. The shorter chain length reduces steric hindrance and acyl migration is fostered compared to long-chain fatty acids (Zhang et al. 2024). Depending on the reaction conditions, the water content in the reaction mixture affects acyl migration in different ways. In our solvent-free system, the high water content seems to enhance acyl migration as observed in a study investigating the enzymatic production of structured phospholipids (Vikbjerg et al. 2005). As MGs were also identified in the spectra, it is evident that some of the DGs are further hydrolyzed by PCI_Lip to produce MGs. With PCI_Lip acting as a putative 1,3-selective lipase, one would expect 2-MG to be the most abundant form of monoglycerides produced from the second stage of hydrolysis. The opposite was observed in our experiments, with 1-MGs being the dominant form of monoglycerides identified in the hydrolysis products (Supplementary Table S6). There are two more theories to explain the presence of 1-MG besides the chance to be a hydrolysis product of the lipase from 1,2-DG, its main product in the first stage of hydrolysis. Firstly, 1-MG might have been a product of the enzymatic hydrolysis of 1,3-DG arising from acyl migration. Secondly, 1-MGs might have originated from 2-MG after acyl migration. Due to the large acyl migration measured under our experimental conditions for 1,2-DGs, the first hypothesis is far stronger, although the contribution of 2-MG acyl migration cannot be ruled out. In the literature, especially 1,2-DG and 2-MG are described to be prone to acyl migration. Therefore, a nucleophilic attack of primary hydroxyl oxygen to the secondary acyl carbon group is proposed. This leads to the formation of an intramolecular ring intermediate, which opens up to the thermodynamically more stable 1,3-DG or 1-MG, respectively (Laszlo et al. 2008). Overall, the investigations on the regioselectivity of PCI_Lip revealed that the enzyme favors the hydrolysis of the outer (*sn* 1,3) esterified fatty acids in the first stage of hydrolysis of a TG. This selectivity makes PCI_Lip an interesting enzyme for the industrial production of tailored lipids, which can be found in infant nutrition. Nevertheless, acyl migration was found to play a major role in this specific setup.

## Conclusion

The optimal support for PCI_Lip immobilization was identified through a comprehensive screening of support materials. Remarkably, five His_6_-tag affinity immobilisates and two adsorption immobilisates exhibited superior binding capacities (15–19 µg protein · (mg carrier)^−1^) with minimal leakage (< 2 µg protein · (mg carrier)^−1^). Recycling studies highlighted IB-HIS-19 as the most suitable support, maintaining 65% of the free lipase’s activity. Consequently, IB-HIS-19 was chosen for in-depth characterization. Intriguingly, immobilization preserved PCI_Lip’s activity and selectivity, whereas immobilization of two double mutants induced notable changes. The double mutant S163M+L302G demonstrated a striking enhancement in activity against medium-chain fatty acids, likely attributable to hyperactivation. Notably, immobilization bolstered the enzyme’s kinetic stability by 5 °C. Further the thermodynamic stability of PCI_Lip was assessed, showing that the lipase has a *T*_M_ of 63 °C. Careful optimization of buffer conditions, concentrations, pH values, and additives unveiled the most stabilizing environments. Citrate buffer and PPB (pH 5.5–7.0) emerged as the most effective stabilizers, with higher concentrations further augmenting thermostability. Salts and sugars were particularly effective in increasing *T*_M_. A combinatorial approach, utilizing 400 mM citrate buffer (pH 6.0) or PPB (pH 7.0) with glycerol, NaCl, glucose, or trehalose, achieved a remarkable increase in *T*_M_ to over 70 °C. Regioselectivity studies revealed that PCI_Lip preferentially targets outer fatty acids in TGs, as evidenced by the predominant formation of 1,2-DG during trioctanoate conversion, as confirmed by ^1^H-NMR analysis.

## Supplementary Information

Below is the link to the electronic supplementary material.

ESM 1 PDF (456 KB)

## Data Availability

Data and materials will be made available on request.
