# Supplementary material for: Investigation of regioselectivity and thermostability of free and immobilized Pleurotus citrinopileatus lipase
Source: Appl Microbiol Biotechnol. 2026 Apr 7;110:134. doi: 10.1007/s00253-026-13801-5 (PMC13056782; doi:10.1007/s00253-026-13801-5)
Supplement: Supplementary file 1 [file 253_2026_13801_MOESM1_ESM.pdf]

# Supplementary material

## Investigation of Regioselectivity and Thermostability of free and immobilized *Pleurotus citrinopileatus* Lipase

Lea Henrich[a], Jan Passinger[a], Moritz Nawrath[a], Foteini Batsolaki[b], Apostolos Spyros[b], Ioannis V. Pavlidis[b], Binglin Li[a,c,d*], Martin Gand[a,*]

[a] Institute of Food Chemistry and Food Biotechnology, Justus Liebig University Giessen, Germany

[b] Department of Chemistry, University of Crete, Heraklion, Greece

[c] College of Food Science and Engineering, Northwest University, Shaanxi, China

[d] Institute of Food Science and Technology Nutrition and Health (Cangzhou), Chinese Academy of Agricultural Sciences, Cangzhou, China

* Corresponding authors

**Supplementary Table S1.** Support materials used in this study with information on the respective matrix, functionalization and polarity as well as the supplier.

| Labeling | Matrix | Functional group | Polarity | Supplier |
|---|---|---|---|---|
| ECR 1090M | macroporous polystyrene | | hydrophobic | Purolite, Ratingen, Germany |
| ECR 8309M | methacrylate | amino $C_2$ | hydrophilic | Purolite |
| ECR 8409M | methacrylate | amino $C_2$ | slightly hydrophilic | Purolite |
| ECR 8806M | methacrylate | octadecyl | hydrophobic | Purolite |
| IB-HIS-1 | polyacryl | low Ni-IDA, high butyl | very hydrophobic | ChiralVision, Den Hoorn, Netherlands |
| IB-HIS-2 | polyacryl | Ni-IDA, low butyl | hydrophilic | ChiralVision |
| IB-HIS-3 | polyacryl | lowNi-IDA, octadecyl | very hydrophobic | ChiralVision |
| IB-HIS-4 | polyacryl | Ni-IDA, hexadeyl/octadecyl | hydrophobic | ChiralVision |
| IB-HIS-7 | polyacryl | Ni-IDA, hydroxyethyl | very hydrophilic | ChiralVision |
| IB-HIS-8 | polyacryl | Ni-IDA | very hydrophilic | ChiralVision |
| IB-HIS-9 | polyacryl | Ni-IDA, ammonia quenched | very hydrophilic | ChiralVision |
| IB-HIS-11 | polyacryl | Ni-NTA, diamine | hydrophilic | ChiralVision |
| IB-HIS-14 | polyacryl | Ni-NTA | hydrophilic | ChiralVision |
| IB-HIS-15 | silica | Ni-NTA, aminopropyl | extremely hydrophilic | ChiralVision |
| IB-HIS-16 | cellulose | Ni-NTA | extremely hydrophilic | ChiralVision |
| IB-HIS-17 | polyacryl | nickel, IDA | hydrophilic | ChiralVision |
| IB-HIS-18 | polyacryl | Ni-NTA, diamine | hydrophobic | ChiralVision |
| IB-HIS-19 | polyacryl | Ni-NTA, diamine | very hydrophiobic | ChiralVision |
| IB-HIS-20 | polyacryl | Ni-NTA, ammonia | very hydrophilic | ChiralVision |
| IB-HIS-21 | silica | Ni-IDA, propyl | extremely hydrophilic | ChiralVision |
| IB-HIS-22 | cellulose | Ni-IDA | extremely hydrophilic | ChiralVision |

**Supplementary Table S2.** Buffers used in the thermofluor screening with the concentrations and pH-values tested. Listed with vendors of the chemicals. pH-values were adjusted by the addition of 1 M HCl or NaOH, respectively.

| Buffer | Concentration [mM] | pH-value | Vendor | Purity |
|---|---|---|---|---|
| citrate | 50, 100, 200, 400 | 4.0, 4.5, 5.0, 5.5, 6.0 | Carl Roth, Karlsruhe, Germany | 99.5% |
| potassium phosphate buffer (PPB) | 50, 100, 200, 400 | 6.0, 6.5, 7.0, 7.5, 8.0 | Carl Roth | ≥ 99% |
| 4-(2-hydroxyethyl)-1-piperazineethanesulfonic acid (HEPES) | 50, 100, 200, 400 | 7.0 | abcr GmbH, Karlsruhe, Germany | 99% |
| 3-(*N*-morpholino)propanesulfonic acid (MOPS) | 50, 100, 200, 400 | 7.0 | Carl Roth | ≥ 99.5% |
| tris(hydroxymethyl)aminomethane (TRIS) | 100, 200, 400 | 7.0, 7.5, 8.0, 8.5, 9.0 | Carl Roth | ≥ 99.3% |

**Supplementary Table S3.** Additives used in the thermofluor screening with the applied concentrations and vendors.

| Additive | Concentration [mM] | Percent by Volume [%, v/v] | Vendor | Purity |
|---|---|---|---|---|
| $(NH_4)_2SO_4$ | 10, 20, 50, 100 | - | Carl Roth, Karlsruhe, Germany | ≥ 99% |
| $CaCl_2$ | 5, 20, 50 | - | Carl Roth | ≥ 99% |
| ethylenediaminetetraacetic acid (EDTA) | 50, 100, 200 | - | Carl Roth | ≥ 99% |
| KCl | 50, 100, 200 | - | CHEMSOLUTE, Renningen, Germany | 99.5% |
| KI | 50, 100, 200 | - | Honeywell Riedel-de Haën, Seelze, Germany | ≥ 99% |
| $MgCl_2$ | 50, 100, 200 | - | AppliChem GmbH, Darmstadt, Germany | ≥ 98.5% |
| NaBr | 50, 100 | - | Carl Roth | ≥ 99% |
| NaCl | 50, 100, 200 | - | VWR International, Leuve, Belgium | ≥ 99.5% |
| NaI | 50, 100, 200 | | Sigma Aldrich, Darmstadt, Germany | ≥ 99% |
| dimethyl sulfoxide (DMSO) | - | 2.5, 5, 10 | Honeywell Riedel-de Haën | 99.9% |
| glycerol | - | 2.5, 5, 10 | VWR International | 99.5% bidestilled |
| polyethylene glycol 20000 (PEG) | - | 2.5, 5, 10 | Carl Roth | Ph. Eur. |
| betaine | 50, 100, 200 | - | Acros Organics, Geel, Belgium | 99% |
| glycine | 50, 100, 200 | - | Carl Roth | 99% |
| proline | 50, 100, 200 | - | Carl Roth | ≥ 98.5% |
| glucose | 50, 100, 200 | - | Carl Roth | ≥ 97.5% |
| trehalose | 50, 100, 200 | - | Carl Roth | 99% |

**Supplementary Table S4.** Lower and upper confidence intervals (95 % CI) and determination coefficient ($R^2$) for the determination of $T_{50}^{60}$ values.

| Variant | Lower CI $T_M$ [°C] | Upper CI $T_M$ [°C] | $R^2$ |
|---|---|---|---|
| free WT | 26.1 | 33.2 | 0.91 |
| immobilized WT | 35.2 | 39.1 | 0.99 |
| free S163M+L302G | 34.7 | 41.2 | 0.97 |
| immobilized S163M+L302G | 42.6 | 44.7 | 0.99 |
| free I245F+L302G | 33.9 | 35.4 | 0.99 |
| immobilized I245F+L302G | 35.3 | 44.0 | 0.99 |

**Supplementary Table S5.** Assignment of signals in the $^{1}H$-NMR spectra of the conversion products of trioctanoate by PCI_Lip WT, the standards and the migration experiments. The chemical shifts and multiplicities are assigned to the types of protons and compounds dissolved in acetone-$D_6$.

| Signal | Chemical Shift [ppm] | Multiplicity | Type of Protons | Compound |
|---|---|---|---|---|
| A | 5.27 | m | $ROCH_2$-C**H**(OR')-$CH_2$OR'' | glceryl group in TG |
| B | 5.07 | m | $ROCH_2$-C**H**(OR')-$CH_2$OH | glyceryl group in 1,2-DG |
| C | 4.86 | m | $HOCH_2$-C**H**(OR)-$CH_2$OH | glyceryl group in 2-MG |
| D1 + D2 | 4.18 + 4.33 | dd, dd | ROC**H₂**-CH(OR')-C**H₂**OR'' | glceryl group in TG |
| E | 4.10 | m | ROC**H₂**-CH(OH)-C**H₂**OR' | glyceryl group in 1,3-DG |
| F | 4.05 | m | $ROCH_2$-C**H**(OH)-$CH_2$OR' | glyceryl group in 1,3-DG |
| G | 3.82 | m | $ROCH_2$-C**H**(OH)-$CH_2$OH | glycery group in 1-MG |
| H | 3.67 | m | $ROCH_2$-CH(OR')-C**H₂**OH | glyceryl group in 1,2-DG |
| I | 3.54 | m | $ROCH_2$-CH(OH)-C**H₂**OH | glyceryl group in 1-MG |

**Table S6.** Amounts of glycerides in percent with half span calculated from duplicates present in the blank and both experiments with incubation of 30 min and 2 h. Glyceride quantification was performed by integrating peaks A, B, C, F and G for TG, 1,2-DG, 2-MG, 1,3-DG and 1-MG, respectively.

| | **triglycerides [%]** | **1,2-diglycerides [%]** | **1,3-diglycerides [%]** | **2-monoglycerides [%]** | **1-monoglycerides [%]** |
|---|---|---|---|---|---|
| blank | 100 ± 0 | 0 ± 0 | 0 ± 0 | 0 ± 0 | 0 ± 0 |
| 30 min incubation | 87.8 ± 0.5 | 6.5 ± 0.2 | 1.2 ± 0.1 | 1.4 ± 0.2 | 3.1 ± 0.6 |
| 2 h incubation | 66.9 ± 3.5 | 12.7 ± 0.8 | 4.4 ± 0.1 | 3.2 ± 0.6 | 13.0 ± 2.2 |

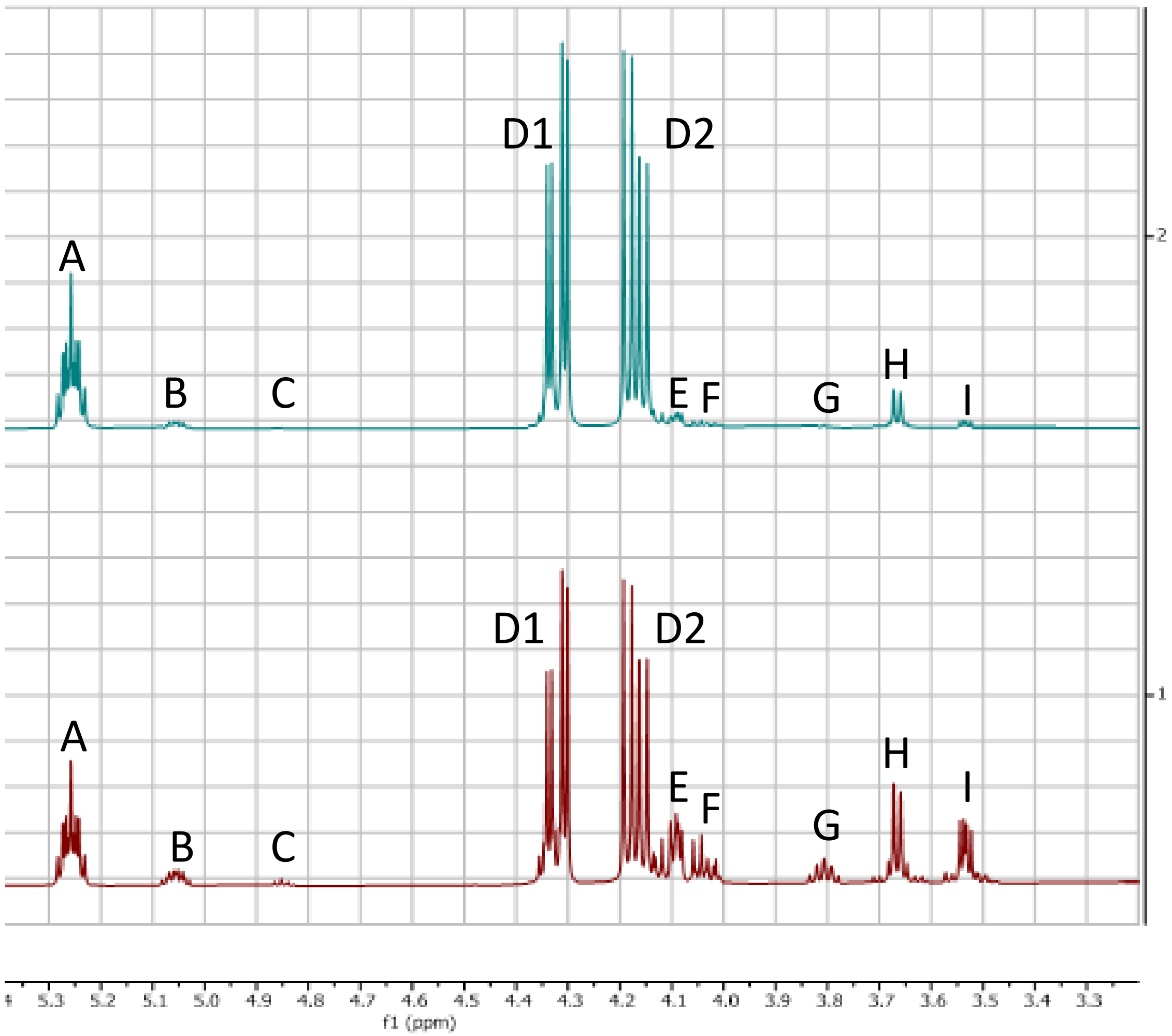


**Supplementary Figure S1**. $^{1}$H-NMR spectra of the conversion products of trioctanoate hydrolyzed with PCI_Lip WT after 30 min of incubation (top, blue) and 2 h of incubation (bottom, red) dissolved in acetone-$D_6$. Assignment of the letters of the signal is given in Supplementary Table S5. Glyceride quantification was performed by integrating peaks A, B, C, F and G for TG, 1,2-DG, 2-MG, 1,3-DG and 1-MG respectively.

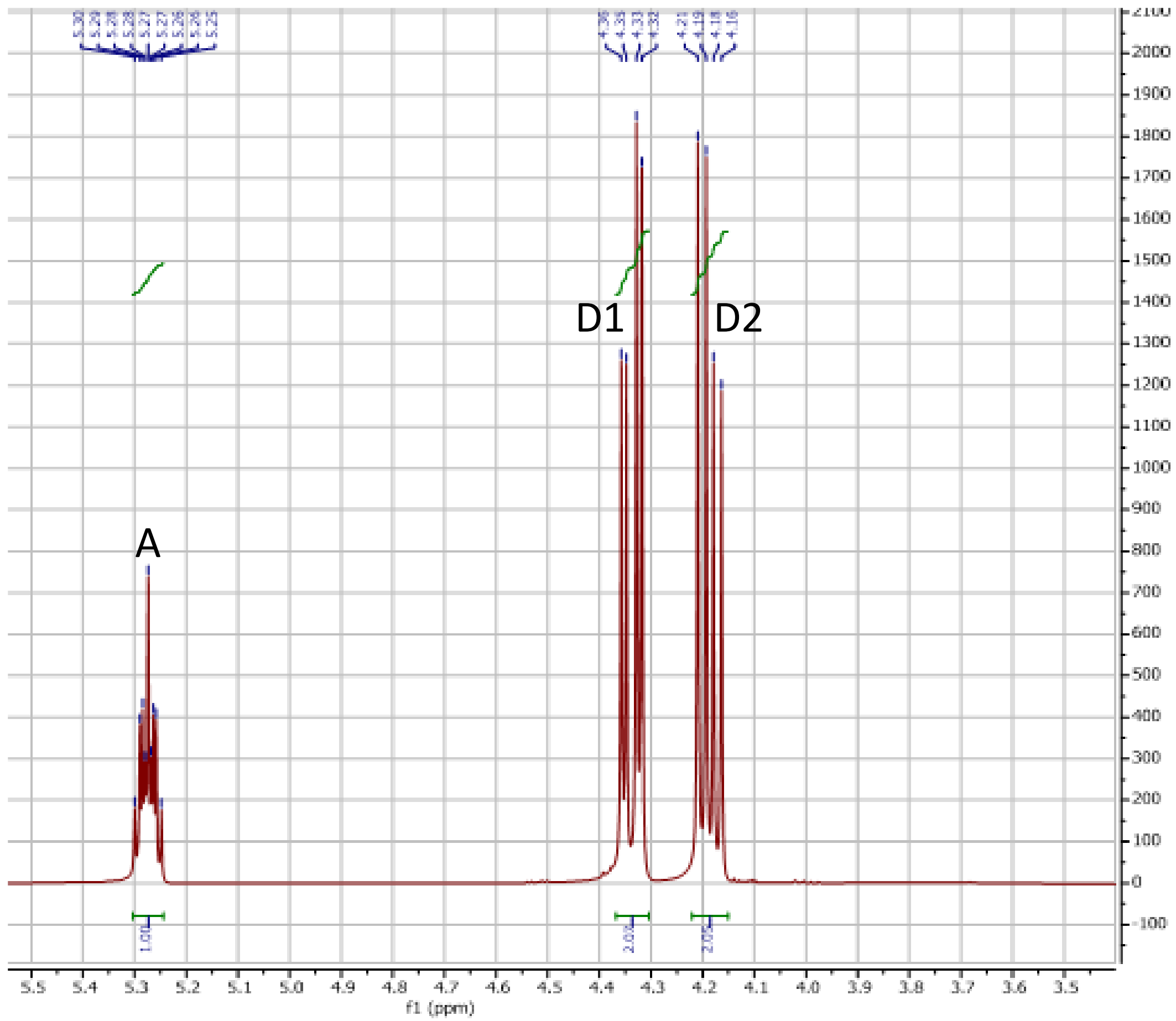


**Supplementary Figure S2.** $^{1}H$-NMR spectrum of the trioctanoate standard dissolved in acetone-$D_6$. Assignment of the letters of the signal is given in Supplementary Table S5.

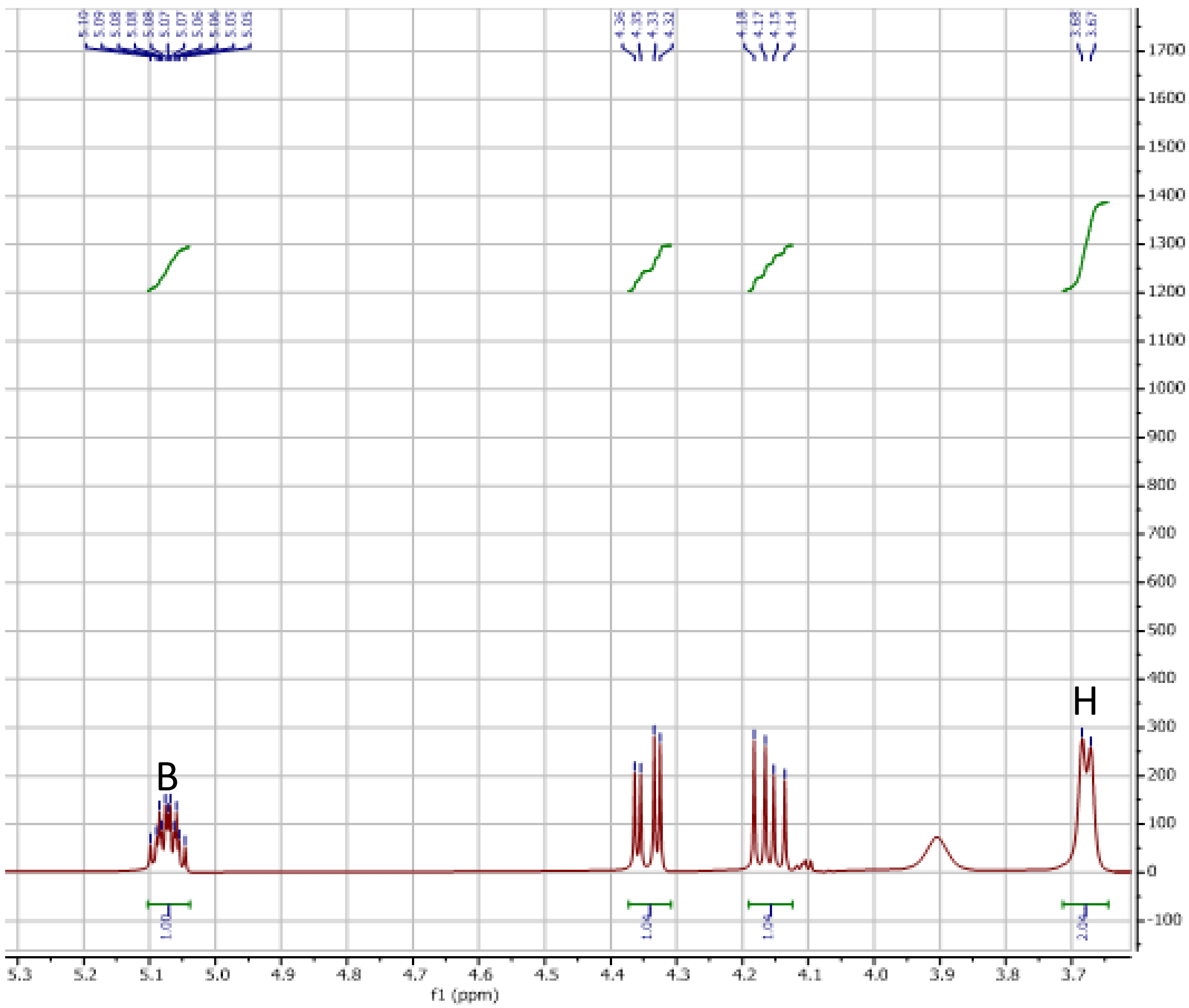


**Supplementary Figure S3.** $^{1}$H-NMR spectrum of the 1,2-dioctanoyl glycerol (1,2-DG) standard dissolved in acetone-$D_6$. Assignment of the letters of the signal is given in Supplementary Table S5.

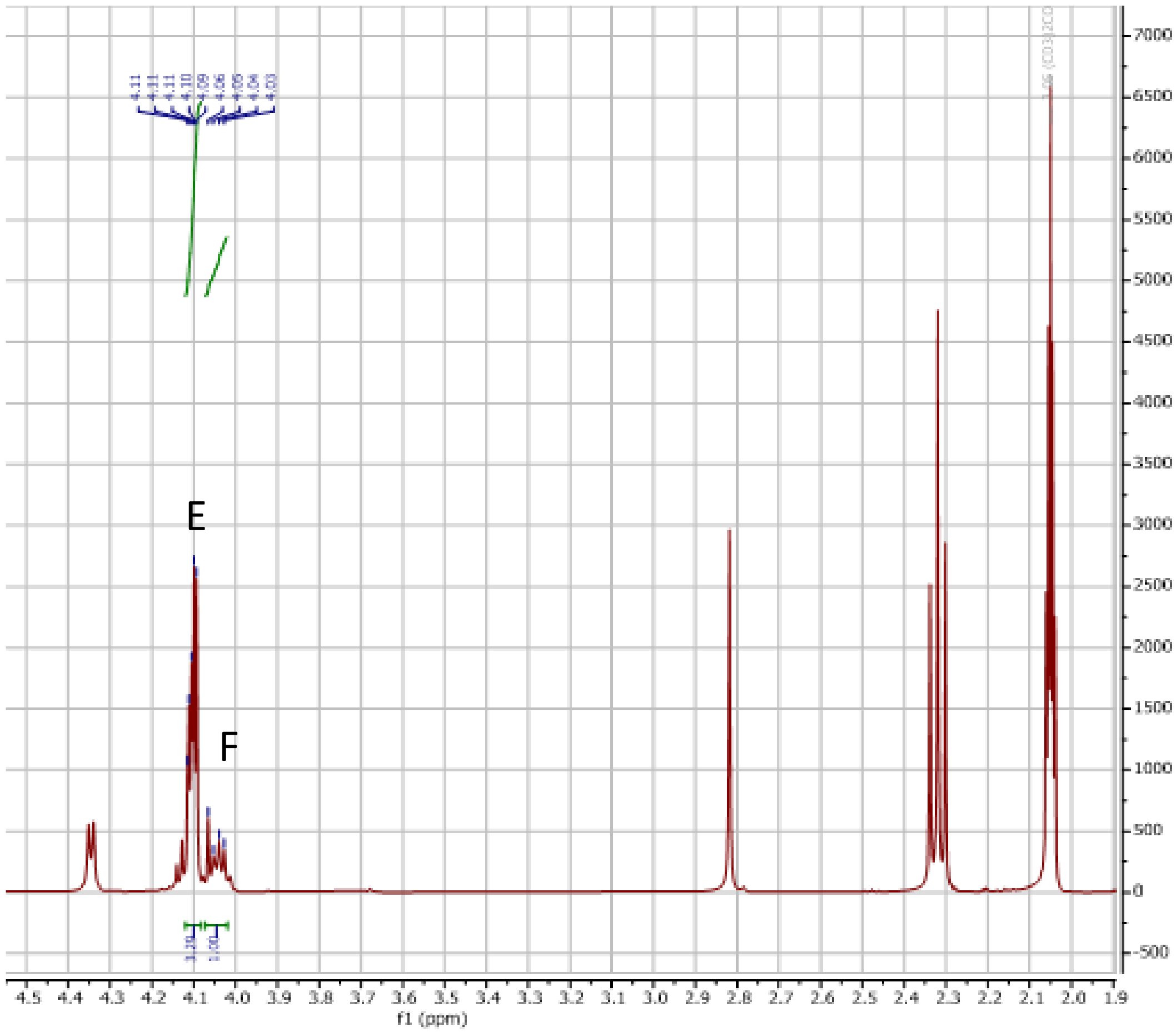


**Supplementary Figure S4.** $^1$H-NMR spectrum of the 1,3-dioctanoyl glycerol (1,3-DG) standard dissolved in acetone-$D_6$. Assignment of the letters of the signal is given in Supplementary Table S5.

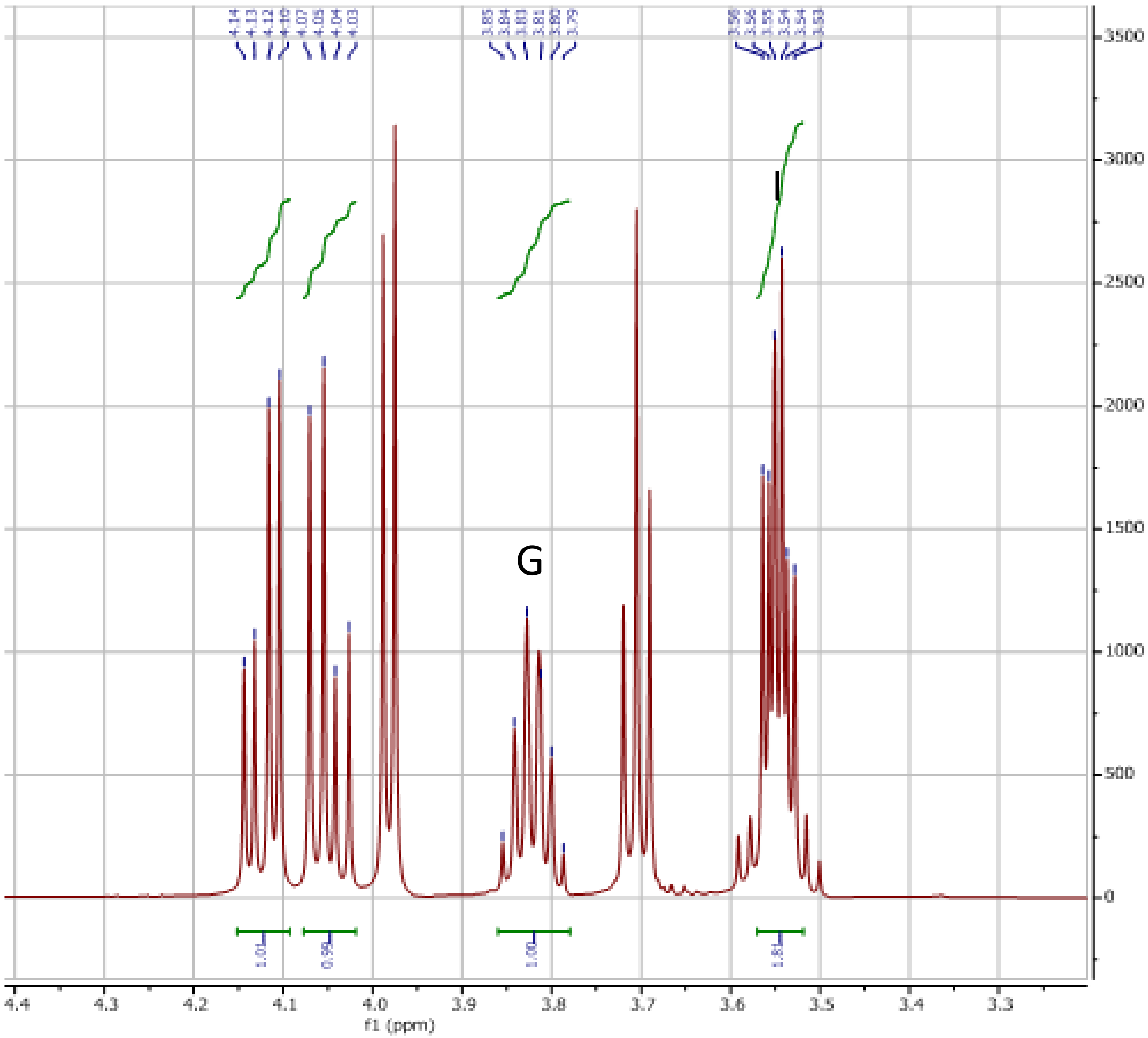


**Supplementary Figure S5.** $^{1}$H-NMR spectrum of the 1-octanoyl glycerol (1-MG) standard dissolved in acetone-$D_6$. Assignment of the letters of the signal is given in Supplementary Table S5.

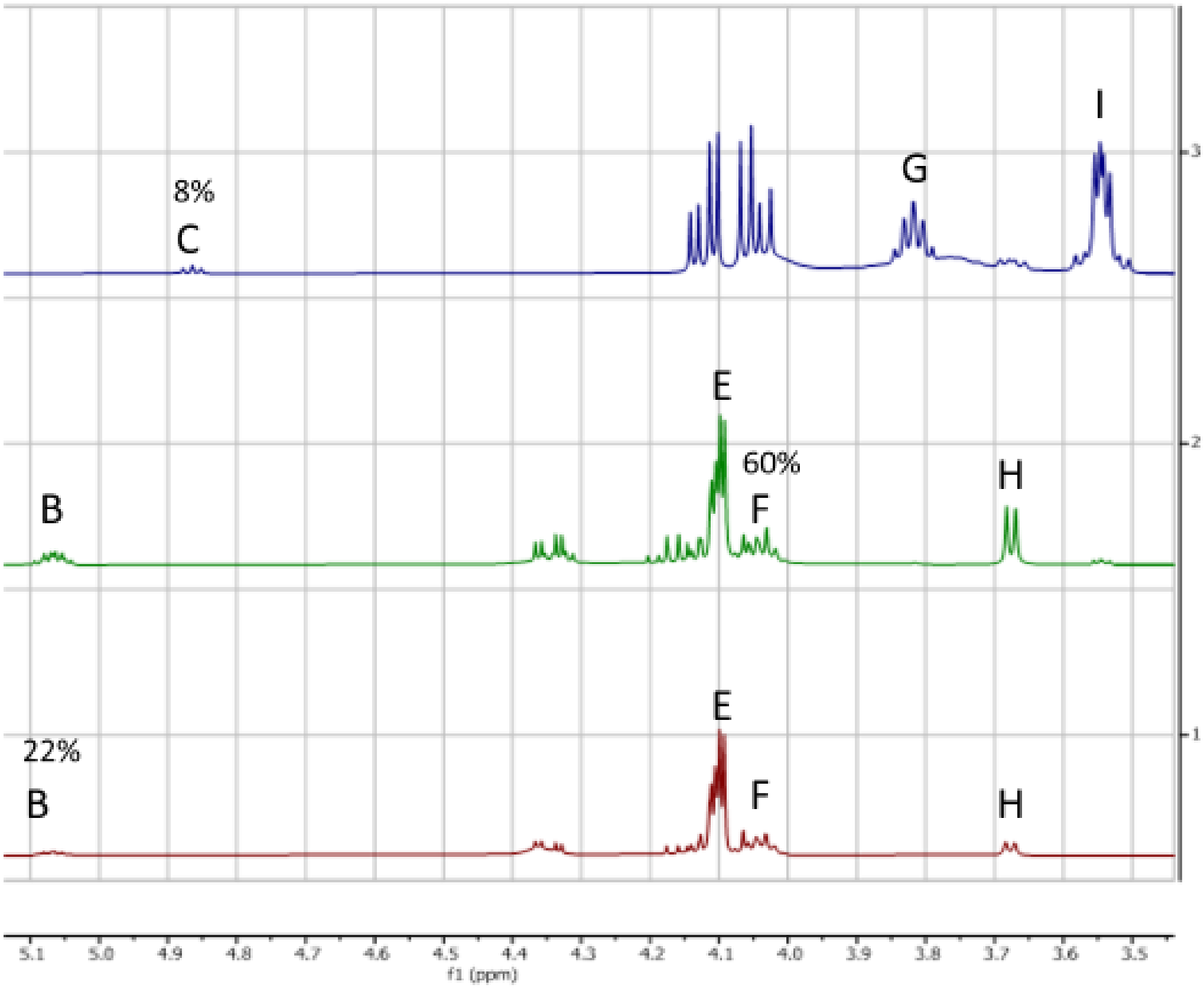


**Supplementary Figure S6.** $^{1}$H-NMR spectra of the products of the acyl migration experiments of 1-MG (top, blue), 1,2-DG (middle, green), and 1,3-DG (bottom, red) dissolved in acetone-$D_6$. Assignment of the letters of the signal is given in Supplementary Table S5. The numbers indicate the % acyl migration calculated from the NMR spectra.
