# Supplementary material for: Investigation of regioselectivity and thermostability of free and immobilized Pleurotus citrinopileatus lipase
Source: Appl Microbiol Biotechnol. 2026 Apr 7;110(1):134. doi: 10.1007/s00253-026-13801-5 (PMC13056782; doi:10.1007/s00253-026-13801-5)
Supplement: Supplementary file 1 — PDF (456 KB) [file 253_2026_13801_MOESM1_ESM.pdf]

Supplementary material

Investigation of Regioselectivity and Thermostability of free and immobilized *Pleurotus citrinopileatus* Lipase

Lea Henrich[a], Jan Passinger[a], Moritz Nawrath[a], Foteini Batsolaki[b], Apostolos Spyros[b], Ioannis V. Pavlidis[b], Binglin Li[a,c,d*], Martin Gand[a,*]

[a] Institute of Food Chemistry and Food Biotechnology, Justus Liebig University Giessen, Germany

[b] Department of Chemistry, University of Crete, Heraklion, Greece

[c] College of Food Science and Engineering, Northwest University, Shaanxi, China

[d] Institute of Food Science and Technology Nutrition and Health (Cangzhou), Chinese Academy of Agricultural Sciences, Cangzhou, China

* Corresponding authors

Supplementary Table S1. Support materials used in this study with information on the respective matrix, functionalization and polarity as well as the supplier.

Labeling	Matrix	Functional group	Polarity	Supplier
ECR 1090M	macroporous polystyrene		hydrophobic	Purolite, Ratingen, Germany
ECR 8309M	methacrylate	amino C_2	hydrophilic	Purolite
ECR 8409M	methacrylate	amino C_2	slightly hydrophilic	Purolite
ECR 8806M	methacrylate	octadecyl	hydrophobic	Purolite
IB-HIS-1	polyacryl	low Ni-IDA, high butyl	very hydrophobic	ChiralVision, Den Hoorn, Netherlands
IB-HIS-2	polyacryl	Ni-IDA, low butyl	hydrophilic	ChiralVision
IB-HIS-3	polyacryl	lowNi-IDA, octadecyl	very hydrophobic	ChiralVision
IB-HIS-4	polyacryl	Ni-IDA, hexadeyl/octadecyl	hydrophobic	ChiralVision
IB-HIS-7	polyacryl	Ni-IDA, hydroxyethyl	very hydrophilic	ChiralVision
IB-HIS-8	polyacryl	Ni-IDA	very hydrophilic	ChiralVision
IB-HIS-9	polyacryl	Ni-IDA, ammonia quenched	very hydrophilic	ChiralVision
IB-HIS-11	polyacryl	Ni-NTA, diamine	hydrophilic	ChiralVision
IB-HIS-14	polyacryl	Ni-NTA	hydrophilic	ChiralVision
IB-HIS-15	silica	Ni-NTA, aminopropyl	extremely hydrophilic	ChiralVision
IB-HIS-16	cellulose	Ni-NTA	extremely hydrophilic	ChiralVision
IB-HIS-17	polyacryl	nickel, IDA	hydrophilic	ChiralVision
IB-HIS-18	polyacryl	Ni-NTA, diamine	hydrophobic	ChiralVision
IB-HIS-19	polyacryl	Ni-NTA, diamine	very hydrophiobic	ChiralVision
IB-HIS-20	polyacryl	Ni-NTA, ammonia	very hydrophilic	ChiralVision
IB-HIS-21	silica	Ni-IDA, propyl	extremely hydrophilic	ChiralVision
IB-HIS-22	cellulose	Ni-IDA	extremely hydrophilic	ChiralVision

Supplementary Table S2. Buffers used in the thermofluor screening with the concentrations and pH-values tested. Listed with vendors of the chemicals. pH-values were adjusted by the addition of 1 M HCl or NaOH, respectively.

Buffer	Concentration [mM]	pH-value	Vendor	Purity
citrate	50, 100, 200, 400	4.0, 4.5, 5.0, 5.5, 6.0	Carl Roth, Karlsruhe, Germany	99.5%
potassium phosphate buffer (PPB)	50, 100, 200, 400	6.0, 6.5, 7.0, 7.5, 8.0	Carl Roth	≥ 99%
4-(2-hydroxyethyl)-1-piperazineethanesulfonic acid (HEPES)	50, 100, 200, 400	7.0	abcr GmbH, Karlsruhe, Germany	99%
3-(*N*-morpholino)propanesulfonic acid (MOPS)	50, 100, 200, 400	7.0	Carl Roth	≥ 99.5%
tris(hydroxymethyl)aminomethane (TRIS)	100, 200, 400	7.0, 7.5, 8.0, 8.5, 9.0	Carl Roth	≥ 99.3%

Supplementary Table S3. Additives used in the thermofluor screening with the applied concentrations and vendors.

Additive	Concentration [mM]	Percent by Volume [%, v/v]	Vendor	Purity
$(NH_4)_2SO_4$	10, 20, 50, 100	-	Carl Roth, Karlsruhe, Germany	≥ 99%
$CaCl_2$	5, 20, 50	-	Carl Roth	≥ 99%
ethylenediaminetetraacetic acid (EDTA)	50, 100, 200	-	Carl Roth	≥ 99%
KCl	50, 100, 200	-	CHEMSOLUTE, Renningen, Germany	99.5%
KI	50, 100, 200	-	Honeywell Riedel-de Haën, Seelze, Germany	≥ 99%
$MgCl_2$	50, 100, 200	-	AppliChem GmbH, Darmstadt, Germany	≥ 98.5%
NaBr	50, 100	-	Carl Roth	≥ 99%
NaCl	50, 100, 200	-	VWR International, Leuve, Belgium	≥ 99.5%
NaI	50, 100, 200		Sigma Aldrich, Darmstadt, Germany	≥ 99%
dimethyl sulfoxide (DMSO)	-	2.5, 5, 10	Honeywell Riedel-de Haën	99.9%
glycerol	-	2.5, 5, 10	VWR International	99.5% bidestilled
polyethylene glycol 20000 (PEG)	-	2.5, 5, 10	Carl Roth	Ph. Eur.
betaine	50, 100, 200	-	Acros Organics, Geel, Belgium	99%
glycine	50, 100, 200	-	Carl Roth	99%
proline	50, 100, 200	-	Carl Roth	≥ 98.5%
glucose	50, 100, 200	-	Carl Roth	≥ 97.5%
trehalose	50, 100, 200	-	Carl Roth	99%

Supplementary Table S4. Lower and upper confidence intervals (95 % CI) and determination coefficient (R^2) for the determination of T_{50}^{60} values.

Variant	Lower CI T_M [°C]	Upper CI T_M [°C]	R^2
free WT	26.1	33.2	0.91
immobilized WT	35.2	39.1	0.99
free S163M+L302G	34.7	41.2	0.97
immobilized S163M+L302G	42.6	44.7	0.99
free I245F+L302G	33.9	35.4	0.99
immobilized I245F+L302G	35.3	44.0	0.99

Supplementary Table S5. Assignment of signals in the ^{1}H-NMR spectra of the conversion products of trioctanoate by PCI_Lip WT, the standards and the migration experiments. The chemical shifts and multiplicities are assigned to the types of protons and compounds dissolved in acetone-D_6.

Signal	Chemical Shift [ppm]	Multiplicity	Type of Protons	Compound
A	5.27	m	$ROCH_2$-C**H**(OR')-CH_2OR"	glceryl group in TG
B	5.07	m	$ROCH_2$-C**H**(OR')-CH_2OH	glyceryl group in 1,2-DG
C	4.86	m	$HOCH_2$-C**H**(OR)-CH_2OH	glyceryl group in 2-MG
D1 + D2	4.18 + 4.33	dd, dd	ROC**H₂**-CH(OR')-C**H₂**OR"	glceryl group in TG
E	4.10	m	ROC**H₂**-CH(OH)-C**H₂**OR'	glyceryl group in 1,3-DG
F	4.05	m	$ROCH_2$-C**H**(OH)-CH_2OR'	glyceryl group in 1,3-DG
G	3.82	m	$ROCH_2$-C**H**(OH)-CH_2OH	glycery group in 1-MG
H	3.67	m	$ROCH_2$-CH(OR')-C**H₂**OH	glyceryl group in 1,2-DG
I	3.54	m	$ROCH_2$-CH(OH)-C**H₂**OH	glyceryl group in 1-MG

Table S6. Amounts of glycerides in percent with half span calculated from duplicates present in the blank and both experiments with incubation of 30 min and 2 h. Glyceride quantification was performed by integrating peaks A, B, C, F and G for TG, 1,2-DG, 2-MG, 1,3-DG and 1-MG, respectively.

	**triglycerides [%]**	**1,2-diglycerides [%]**	**1,3-diglycerides [%]**	**2-monoglycerides [%]**	**1-monoglycerides [%]**
blank	100 ± 0	0 ± 0	0 ± 0	0 ± 0	0 ± 0
30 min incubation	87.8 ± 0.5	6.5 ± 0.2	1.2 ± 0.1	1.4 ± 0.2	3.1 ± 0.6
2 h incubation	66.9 ± 3.5	12.7 ± 0.8	4.4 ± 0.1	3.2 ± 0.6	13.0 ± 2.2

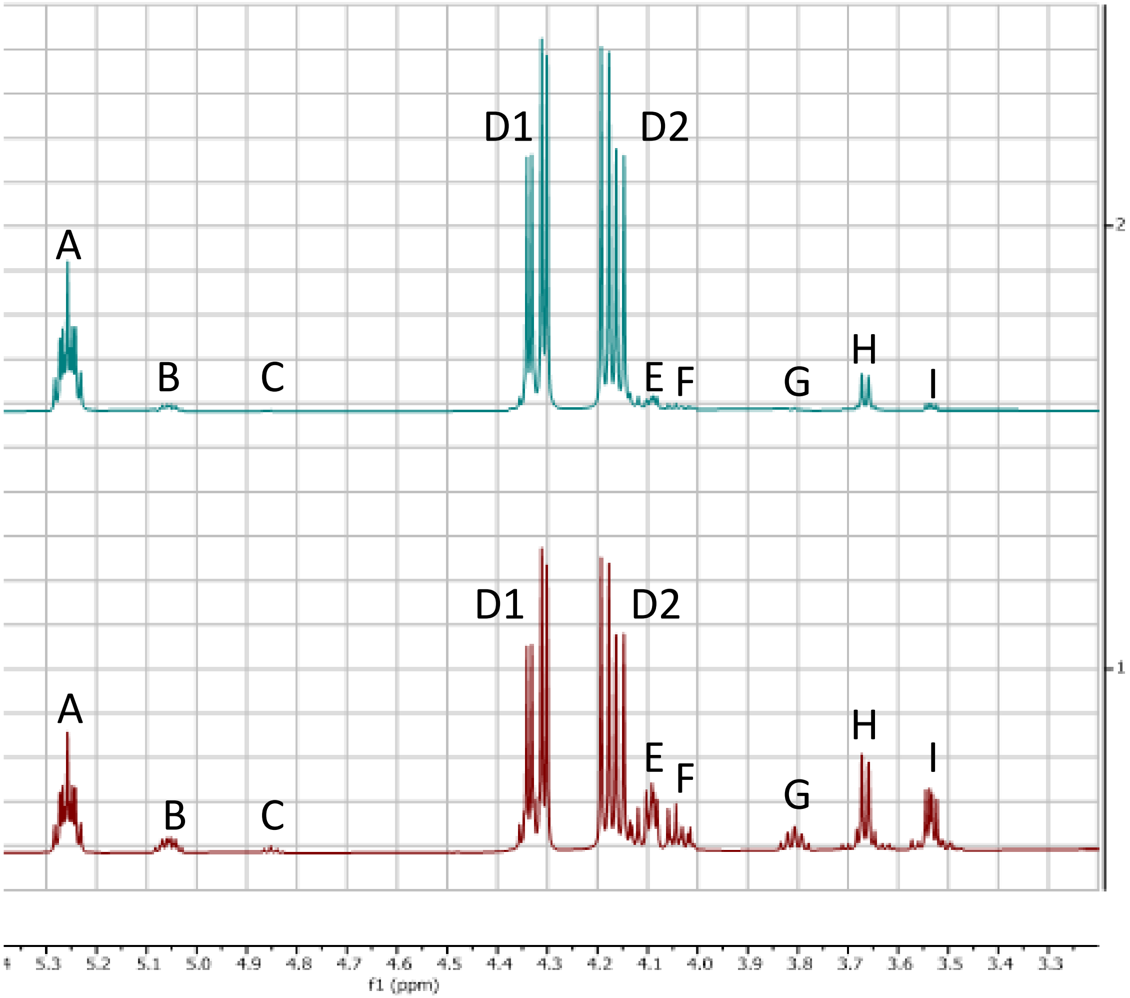

Supplementary Figure S1. ^{1}H-NMR spectra of the conversion products of trioctanoate hydrolyzed with PCI_Lip WT after 30 min of incubation (top, blue) and 2 h of incubation (bottom, red) dissolved in acetone-D_6. Assignment of the letters of the signal is given in Supplementary Table S5. Glyceride quantification was performed by integrating peaks A, B, C, F and G for TG, 1,2-DG, 2-MG, 1,3-DG and 1-MG respectively.

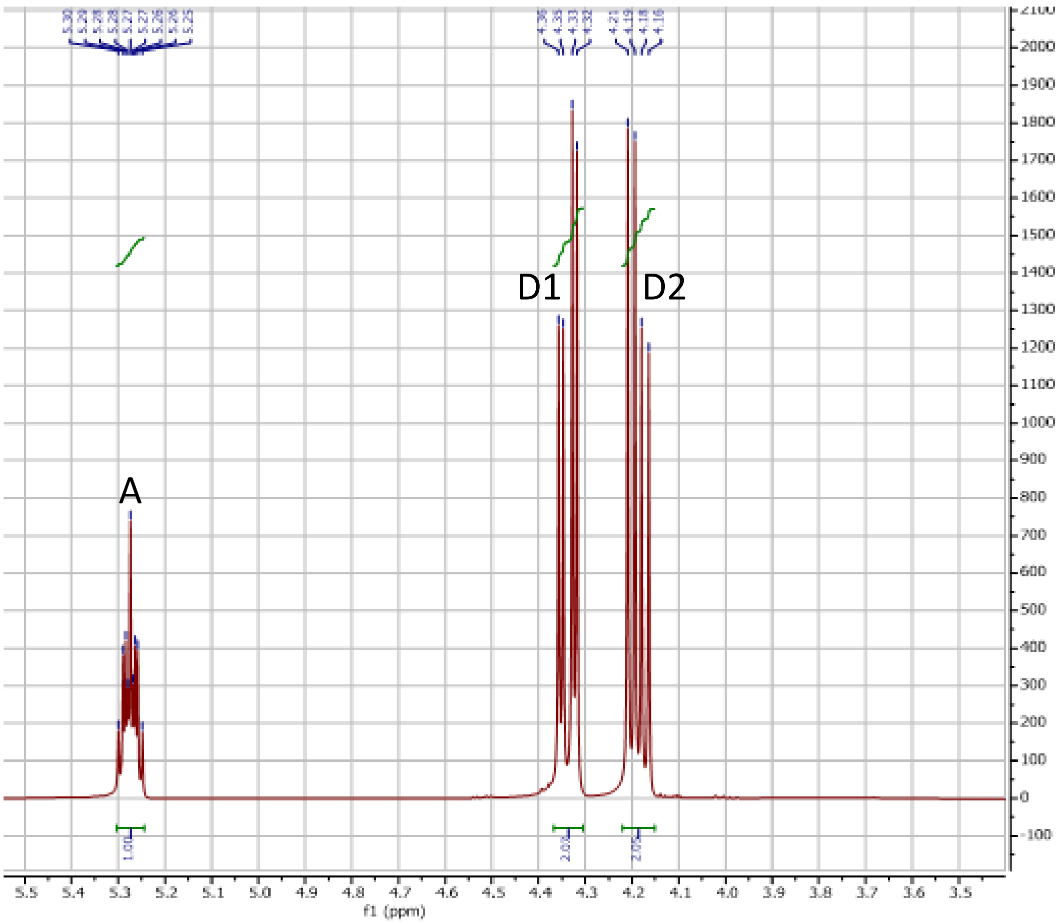

Supplementary Figure S2. ^{1}H-NMR spectrum of the trioctanoate standard dissolved in acetone-D_6. Assignment of the letters of the signal is given in Supplementary Table S5.

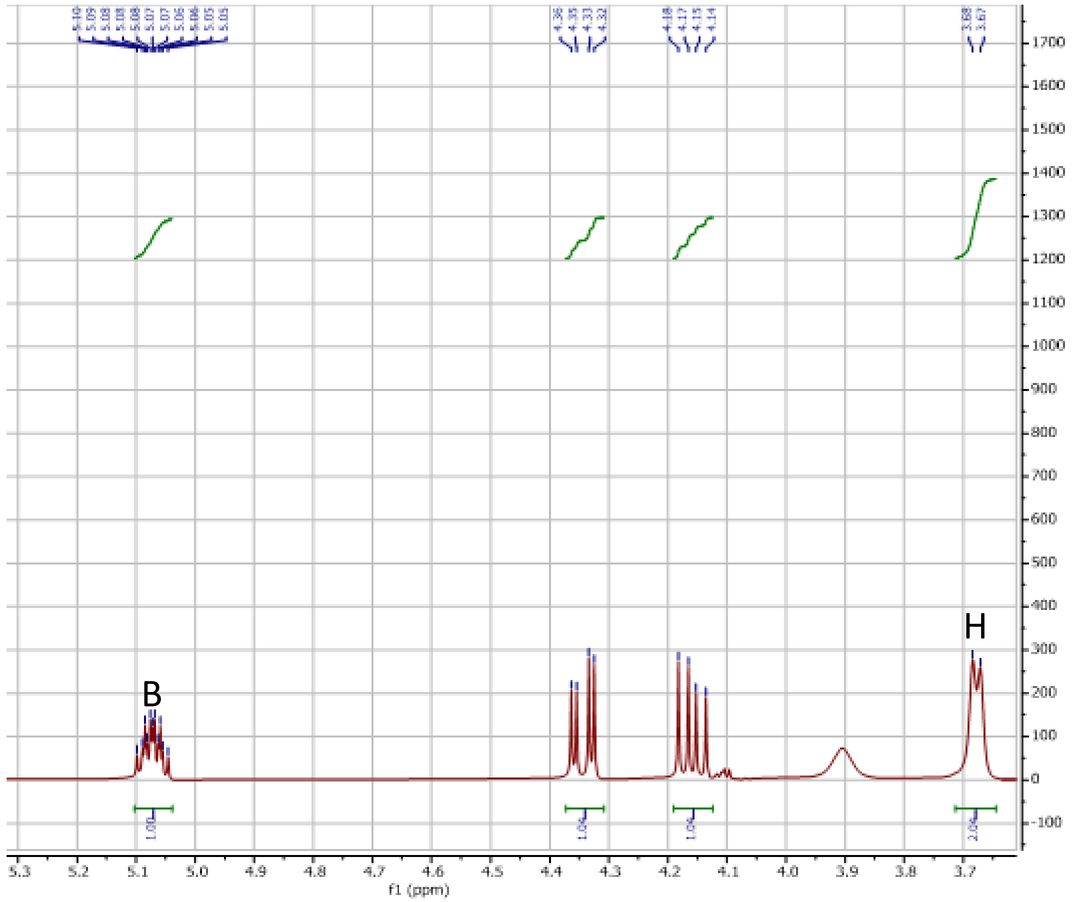

Supplementary Figure S3. ^{1}H-NMR spectrum of the 1,2-dioctanoyl glycerol (1,2-DG) standard dissolved in acetone-D_6. Assignment of the letters of the signal is given in Supplementary Table S5.

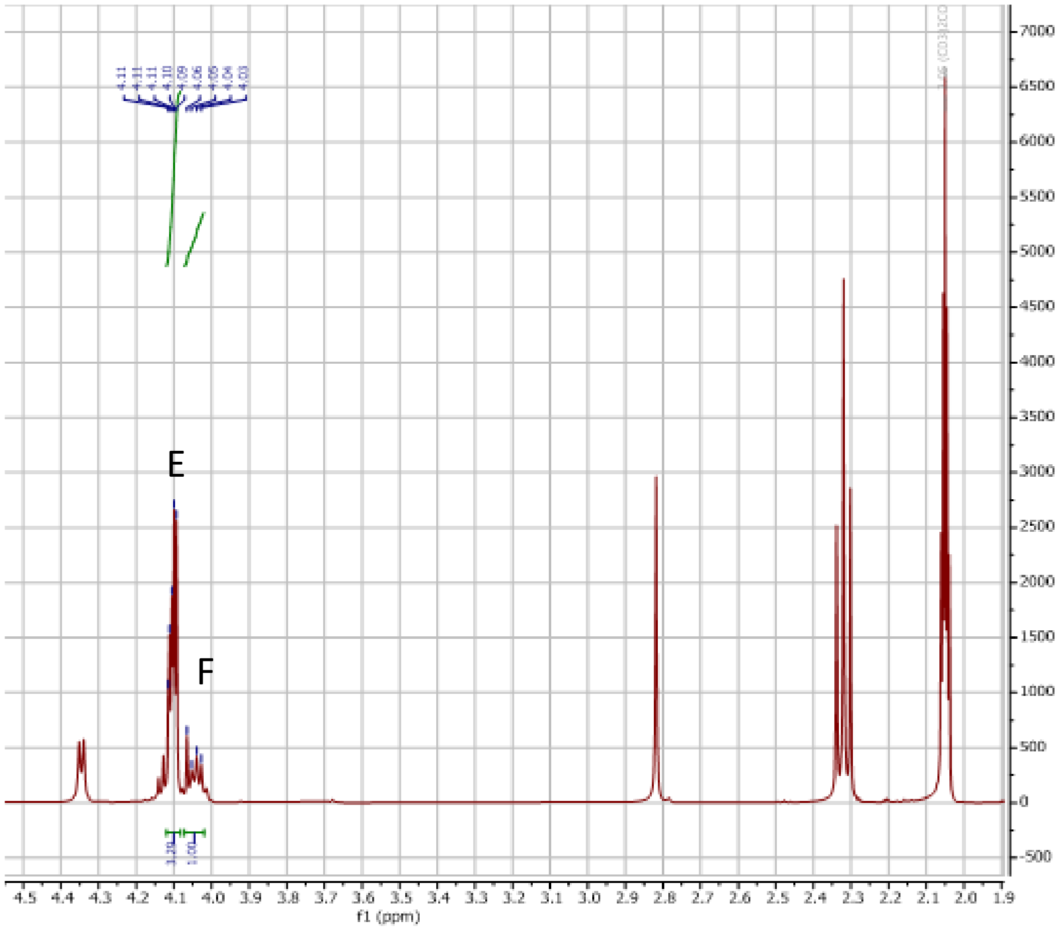

Supplementary Figure S4. ^{1}H-NMR spectrum of the 1,3-dioctanoyl glycerol (1,3-DG) standard dissolved in acetone-D_6. Assignment of the letters of the signal is given in Supplementary Table S5.

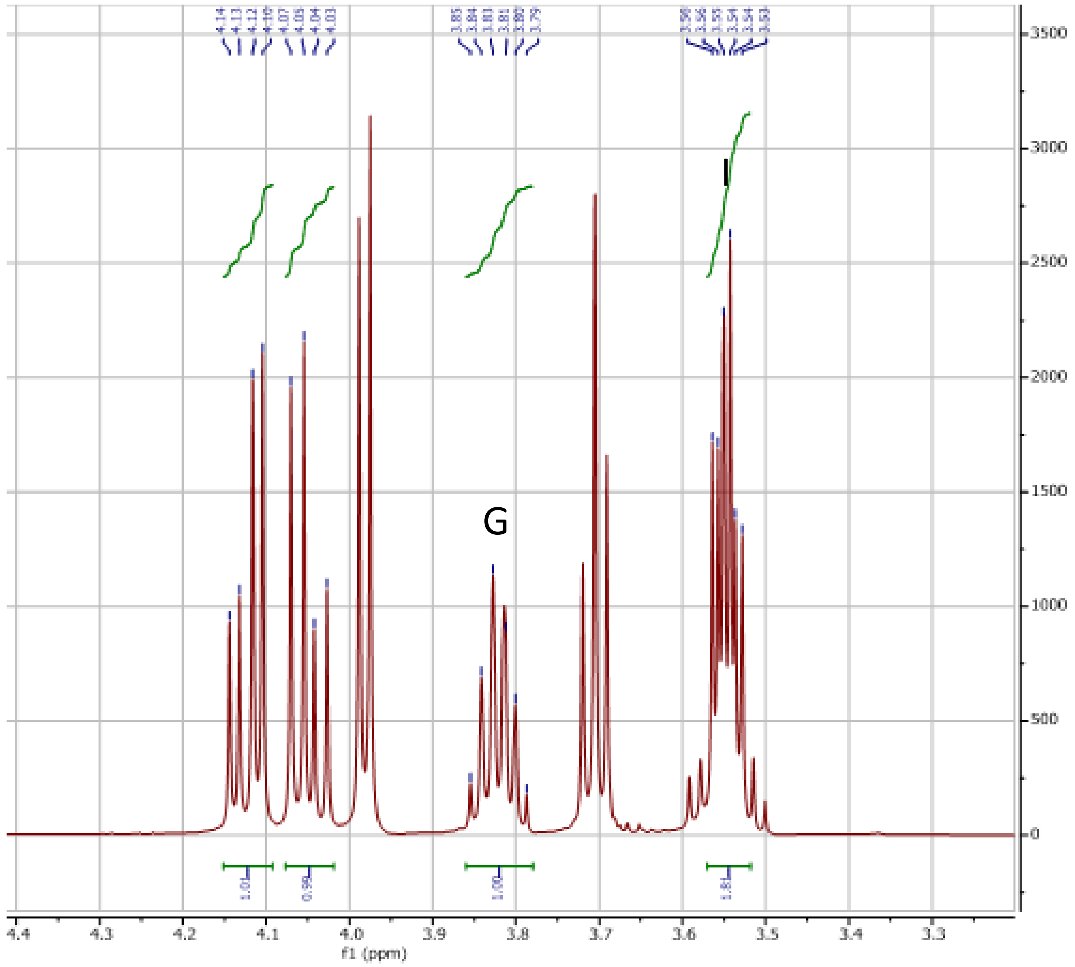

Supplementary Figure S5. ^{1}H-NMR spectrum of the 1-octanoyl glycerol (1-MG) standard dissolved in acetone-D_6. Assignment of the letters of the signal is given in Supplementary Table S5.

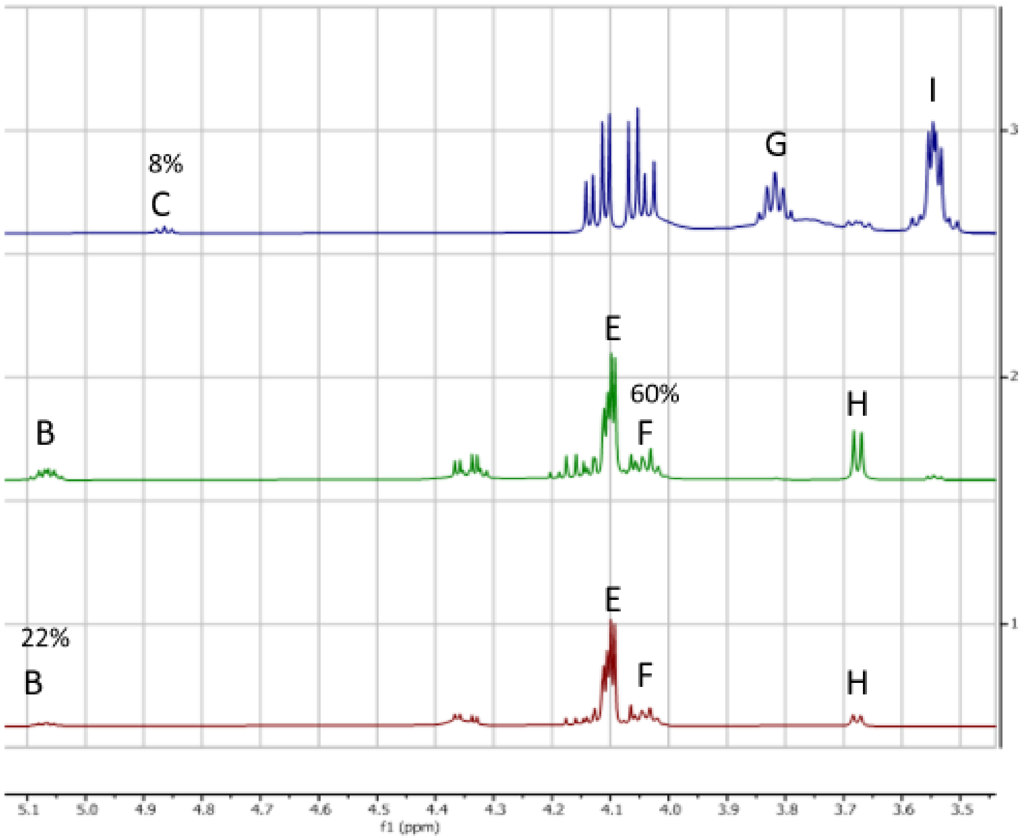

Supplementary Figure S6. ^{1}H-NMR spectra of the products of the acyl migration experiments of 1-MG (top, blue), 1,2-DG (middle, green), and 1,3-DG (bottom, red) dissolved in acetone-D_6. Assignment of the letters of the signal is given in Supplementary Table S5. The numbers indicate the % acyl migration calculated from the NMR spectra.
